# Pectin Methylesterase and Pectin Remodelling Differ in the Fibre Walls of Two *Gossypium* Species with Very Different Fibre Properties

**DOI:** 10.1371/journal.pone.0065131

**Published:** 2013-06-05

**Authors:** Qinxiang Liu, Mark Talbot, Danny J. Llewellyn

**Affiliations:** Plant Industry, Commonwealth Scientific and Industrial Research Organisation (CSIRO), Canberra, Australian Capital Territory, Australia; Lawrence Berkeley National Laboratory, United States of America

## Abstract

Pectin, a major component of the primary cell walls of dicot plants, is synthesized in Golgi, secreted into the wall as methylesters and subsequently de-esterified by pectin methylesterase (PME). Pectin remodelling by PMEs is known to be important in regulating cell expansion in plants, but has been poorly studied in cotton. In this study, genome-wide analysis showed that *PME*s are a large multi-gene family (81 genes) in diploid cotton (*Gossypium raimondii*), an expansion over the 66 in *Arabidopsis* and suggests the evolution of new functions in cotton. Relatively few *PME* genes are expressed highly in fibres based on EST abundance and the five most abundant in fibres were cloned and sequenced from two cotton species. Their significant sequence differences and their stage-specific expression in fibres within a species suggest sub-specialisation during fibre development. We determined the transcript abundance of the five fibre *PMEs*, total PME enzyme activity, pectin content and extent of de-methylesterification of the pectin in fibre walls of the two cotton species over the first 25–30 days of fibre growth. There was a higher transcript abundance of fibre-*PME*s and a higher total PME enzyme activity in *G. barbadense (Gb)* than in *G. hirsutum (Gh)* fibres, particularly during late fibre elongation. Total pectin was high, but de-esterified pectin was low during fibre elongation (5–12 dpa) in both *Gh* and *Gb*. De-esterified pectin levels rose thereafter when total PME activity increased and this occurred earlier in *Gb* fibres resulting in a lower degree of esterification in *Gb* fibres between 17 and 22 dpa. *Gb* fibres are finer and longer than those of *Gh*, so differences in pectin remodelling during the transition to wall thickening may be an important factor in influencing final fibre diameter and length, two key quality attributes of cotton fibres.

## Introduction

The primary cell walls (PCWs) surrounding all dicot plant cells are composed of cellulose microfibrils complexed with interconnecting xyloglucan polymers that are all embedded in a pectin polymer matrix [1]. The pectic matrix provides an environment for the deposition, slippage and extension of the structural cellulosic-glycan network during cell growth, regulates cell wall porosity and is the major adhesive material between adjacent cells [2]. The porosity properties of the matrix influence hydration status and affect the movement and access of cell wall-modifying proteins that interact with the microfibrils and other polymers and proteins in the wall [2].

Pectins are highly complex polymers mainly comprising homogalacturonan (HGA) and rhamnogalacturonans I (RG-I) and II (RG-II) [3], [4]. HGA is a linear homopolymer of (1→4)-α-d-galacturonic acid containing 100–200 galacturonic acid (GalA) residues [5]. HGA is synthesized in the Golgi with 70–80% of the GalA residues methylesterified at the C-6 carboxyl position and then deposited into the cell wall [6]. In addition to HGA, an acidic pectic domain consisting of as many as 100 repeats of the disaccharide (1→2)-α-L-rhamnose-(1→4)-α-D-galacturonic acid is also synthesized and is known as RG-I [2]. Although RG-II is not structurally related to RG-I, it has a backbone of around 9 GalA residues that are (1→4)-α-linked and is substituted by up to 4 heteropolymeric side chains [7].

After pectins are secreted into the cell wall, they can be subsequently modified by pectin methylesterase (PME, EC 3.1.1.11), which catalyses the de-methylesterification of homogalacturonans by converting methoxyl groups into carboxyl groups and releasing both methanol and protons (reviewed in [8]). It appears that most plant PMEs de-esterify the HGA backbone in a progressive or block-wise fashion, resulting in gelation of the pectin and cell wall stiffening due to the formation of localised Ca^2+^ cross-links between the free carboxyl groups of adjacent pectin chains [9]. It has been reported that blocks in the region of 14 contiguous residues are needed for effective calcium cross-linking of HGA chains [10]. Some plant PMEs, however, may have a more random pattern of de-methylesterification, similar to bacterial and fungal PMEs [11]. The pectin modified by those enzymes can also form a calcium-mediated gel, but have more elasticity, compressive and porosity properties that are different from those formed from pectin with linear blocks of de-methylesterification [11]. In addition, the enzymatic action of PME lowers the pH of the cell wall and this has been proposed to regulate the activity of other cell wall modifying enzymes (polygalacturonases, β-glalactosidases, pectate lyases etc.) that have an optimal activity at low pH and thereby facilitates cell expansion and growth [12], cell wall softening and cell separation [13].

Given these diverse functional attributes of de-esterified pectin it is not surprising that PMEs have been reported to be involved in many physiological processes where they can either promote or inhibit cell expansion or wall remodelling such as in fruit ripening [14], pollen tube growth [15], cambial cell differentiation [16], hypocotyl elongation [17] and at the sites of incipient organ development in meristems [18]. Inhibition of the expression of a PME during pea root development, for example, resulted in reduced root elongation, altered root cell morphology and reduced root cap cell separation [19]. While over-expression of a *Petunia* PME in potatoes led to enhanced elongation in shoot apical tissues compared with wild type, but overall tuber yield was reduced [20]. Either increasing or reducing *PtPME1* expression in transgenic lines of poplar was shown to increase or decrease the length of wood fibres, respectively [21]. In *Arabidopsis*, five different PME encoding genes are highly expressed in the xylem, while a dozen PME genes have been found in poplar wood-forming tissues suggesting that specific isoforms might have different functions at different stages during the course of cell differentiation [22].

Cotton (*Gossypium hirsutum* L. and *G. barbadense* L.) fibre development has been considered as a useful simple model system to investigate both cellulose biosynthesis and cell expansion and elongation [23]. The commercial cotton fibre derives from a single cell of the outer epidermis of the ovule that initiates at or just before anthesis, with about 30% of the seed coat epidermal cells developing into these specialized seed hairs or trichomes. Cotton fibre development consists of four overlapping developmental stages: fibre initiation, cell elongation, secondary wall deposition and maturation [24]. After initiation and rapid elongation for some 20 days, thick layers of secondary cell wall (SCW) cellulose is synthesized and deposited under the PCW from about 17 to 40 dpa [25] and constitutes about 96% of the weight of the final dried fibre. The PCW is therefore important in determining the length and diameter of the fibre while the SCW its strength and maturity, all critical parameters in determining the commercial value of cotton fibre for textile manufacture [26]. Since *G. barbadense* species have the longest, finest and strongest fibres of any cotton species, comparative studies between it and *G. hirsutum*, the more commonly grown species, are a good single cell model for investigating the role of PME isoforms in pectin remodelling during cotton fibre elongation and consequently how this might affect cotton fibre quality.

In this study, we have investigated the genomic diversity of different PME genes in cotton, cloned five fibre-expressed cotton *PME* genes and investigated their temporal expression levels during fibre development in two species of cotton. The genes encoded proteins with different structural attributes and expression patterns suggesting they play different roles at different times in fibre development. We also measured total PME enzyme activity and characterised the extent of pectin methylesterification in the pectin extracted from fibre cell walls of both species. Our results demonstrated that there were substantial differences in pectin amount and degree of esterification (DE) at different stages of fibre development within a species, but there were also differences between species in PME expression levels, enzyme activity and DE that are likely to have consequences on the extensibility of the fibre wall and influence the fibre’s physical dimensions and quality.

## Materials and Methods

### Plant Materials

*G. hirsutum* L. cv. Coker 315 and *G. barbadense* L. cv. Pima S7 plants were grown in glasshouse at 30°C/22°C (16 h day/8 h night) and natural lighting supplemented with artificial lamps during the winter months. These two cultivars were chosen because of their large differences in fibre quality attributes. Under field conditions the Coker 315 variety had fibre length, and strength values of 30.48 mm, 29.7 gm/tex respectively, while the Pima S7 fibres were 35.3 mm, 44.3 g/tex as determined by the Uster High Volume Instrument (HVI) (Uster Technologies, http://www.uster.com/en/instruments/fiber-testing/). Flowers of the glasshouse plants were tagged on the day of anthesis and bolls harvested at various times and snap-frozen in liquid nitrogen. Fibres 10 dpa and older were separated from seeds and ground on liquid nitrogen for cell wall and gene expression analysis. The 0, 2 and 5 dpa samples were from the whole ovules with attached fibres.

### *PME* Gene Cloning

After blasting (tBlastn) *Gossypium* species EST databases on NCBI with the sequences of PME proteins from *Arabidopsis* (UniProtKB/Swiss-Prot: Q42534 (PME2_ARATH) and Q9LVQ0 (PME31_ARATH) that are representative of the large and smaller MW type I and II PMEs found in the *Arabidopsis* genome [27]), the sequences putatively encoding pectin methylesterases were identified. ESTs were then assembled in Contig Express in Vector NTi 11.5 software (Invitrogen, Melbourne, Australia) and 41 consensus sequences generated. These were checked for open reading frames and queried against the non-redundant protein database to confirm that they were indeed pectin methylesterases. Thirty three were confirmed but only five contained ESTs from cotton fibres. Five pairs of oligonucleotide primers (Table S2) were initially designed from these consensus sequences (Figure S1) and used to amplify the genes from Coker 315 and Pima S7 cotton fibre cDNA (pooled 0–30 dpa). Five partial putative PME cDNA sequences were initially obtained designated as *GbPME1*, *GbPME2*, *GbPME3, GbPME4* and *GbPME5.* These sequences were extended to full length using a GeneRacer Kit according to the manufacturer’s instructions (Invitrogen, Australia). The equivalent genes were also cloned from *Gh* Coker 315 fibre cDNA. Real time PCR primers were designed mostly from the 3′ untranslated region in such a way that each amplicon would be specific to that gene, but was conserved between the two cotton species of *G. hirsutum* and *G. barbadense* and the two homoeologous genes from the sub-genomes within the species. The presence of signal peptides or signal anchors was analysed using SignalP V3.0 (http://www.cbs.dtu.dk/services/SignalP/). Transmembrane domains were predicted with TMHMM V2.0 (http://www.cbs.dtu.dk/services/TMHMM/).

Sixty six putative *Arabidopsis* PME protein sequences [28] were retrieved from the TAIR Carbohydrate esterase family 8 repository http://www.arabidopsis.org/browse/genefamily/CarbohydrateEsterase.jsp and aligned with the cotton PME protein sequences using ClustalW within the MEGA V5.0 software package (Molecular Evolutionary Genetics Analysis, [29]). *G. raimondii* PME/PMEI sequences were retrieved from the draft genome assembly at http://www.phytozome.net/search.php?show=blast&targetType=genome&method=Org_Graimondii using TBLASTN with default settings and AtPME2 and AtPME31 sequences as search terms. A Phylogenetic tree was drawn with MEGAV5.0 using the neighbour-joining method with complete deletion; 1,000 replicates were used for bootstrap analysis and the cut-off value was 50%.

### RNA Isolation and cDNA Synthesis

Total RNA was isolated from 0, 2 and 5 dpa whole ovules and 10 to 30 dpa isolated fibres which were preserved in RNAlater (Ambion, http://ambion.com) solution as described in [30] and digested with RNase-free TURBO DNAse (Ambion, http://ambion.com) according to the manufacturer’s recommendations. A total of 1 µg of RNA was reverse-transcribed from an oligo(dT)_18_ primer using Superscript III according to the manufacturer’s recommendations (Invitrogen).

### Real-time PCR Experiments

The cDNA templates were diluted 100 times prior to amplification. Real-time PCR was carried out in an Applied Biosystems 7900HT Fast Real-time PCR system (Foster City, CA, USA) according to the following procedure. A 15 µl aliquot of a master mix consisting of 10 µl of 2 × SYBR Green JumpStart Taq Ready Mix (Sigma), 0.5 µl of each 20 µM forward and reverse oligonucleotides corresponding to a given target gene and 4 µl of PCR-grade water were pipetted into 96- or 384- well plates. The templates (5 µl) were then added to the master mixes and transferred to the thermal cycler. Cycling conditions were 5 min of denaturation at 95°C followed by 40 cycles of 95°C denaturation for 15 s, 60°C annealing for 15 s and 72°C elongation for 20 s. Following amplification, a dissociation stage was carried out to detect any complex products as recommended. Data analysis was performed with RQ Manager software (Applied Bioscience) and transcript abundance determined relative to the cotton ubiquitin gene (accession no. EU604080, Table S2) as an internal reference using the ΔCt method.

### Cotton PME Enzyme Assay

Total PME enzyme activity was measured according to [31]. Crude protein extracts were generated from separated fibres of Pima S7 and Coker 315 varieties at various times after anthesis. The collected samples frozen in liquid nitrogen were ground and suspended in 50 mM phosphate buffer pH 7.5. After centrifugation at 12,000×g for 15 min, the supernatants were collected for enzyme assay. The reaction mixture (1 ml) contained 50 mM phosphate buffer, pH 7.5, 0.4 mM NAD, 0.5% (w/v) pectin (from citrus peel, P9135, Sigma), 0.35 U formaldehyde dehydrogenase (from *Pseudomonas putida*, F1879, Sigma), and 1.0 U alcohol oxidase (from *P. pastoris*, A2404, Sigma). After mixing, the reaction was started by the addition of 10 µl crude protein extract from cotton fibres. The reaction mixture was incubated at 37°C for 1 hour and reaction rates were recorded at 340 nm in a Hitachi U-2000 spectrophotometer. One unit of PME activity was defined as 1 µmol NADH/mg protein/minute at 37°C.

### Analysis of Pectin Methylesterification

#### Pectin extraction

Crude pectins were extracted from separated fibres of Pima S7 and Coker 315 varieties as described in [32]. Briefly, the collected fibres were ground in liquid nitrogen and then suspended in 50 mM sodium acetate, pH 5 and incubated for half an hour. After centrifugation at 12,000×g for 15 min, the supernatant was transferred to a fresh tube; ethanol was added to the supernatant to 80% to precipitate the pectin. The suspension was centrifuged at 12,000×g for 15 min. The pellet was then dissolved in water for determinations of pectin content and extent of pectin methylesterification as described below.

#### Pectin content and pectin methylesterification determinations

Polygalacturonate content in cotton fibres was determined by an enzymatic method adapted from [33]. This method is specific for measurement of polygalacturonate and also allows the calculation of the degree of methylesterification of the cell wall pectin, by measuring the percentage of methylesterified galacturonic residues relative to the total amount of polygalacturonates. This method involved a number of sequential enzymatic reactions. Non-methylesterified pectin (2 mg in 200 µl) was first degraded by the addition of two unit of commercial polygalacturonase (PG, Sigma-Aldrich, Sydney, Catalogue No. P-5079) in 50 mM Na_2_PO_3,_ pH 7.5 and incubated for one hour. Aliquot of the reaction mixture (100 µl) was used for assaying the released uronic acid residues according to the uronic acid assay method. The quantitative measurement of uronic acid was carried out by the use of the naphthoresorcinol method [34]. To 400 µl of pectin solution, 0.02 ml of the 19% HCl was added and mixed, then placed in a water bath at 75°C for 45 min. After adding 0.4 ml of concentrated HCl and 0.25 ml of naphthoresorcinol (10% dissolved in 95% ethanol), the tube was allowed to stand for 60 min in a water bath at 50°C. The solution was extracted with 0.8 ml of ether after cooling to room temperature. The two layers were separated by centrifugation at 12,000×g for 5 min and the absorbance of the upper phase was measured at 570 nm. Galacturonic acid (Sigma-Aldrich, Sydney, Australia) was used for the construction of a standard curve and amount of uronic acid from unknown samples was calculated after reference to the standard curve. The remaining reaction mixture containing methyl-esterified pectin regions, which are resistant to polygalacturonase degradation, were treated by the addition of one unit of commercial PME from orange peel (Sigma-Aldrich, Sydney, Catalogue No. P5400) to strip off methyl groups over an incubation of 1 h. The stripped polygalacturonates were degraded with the further addition of one unit of polygalacturonase. The amount of the uronic acid released in the second hydrolysis was therefore a measure of the amount of methylesterified pectin in the original sample. Liberated uronic acid was measured colorimetrically as described above in the naphthoresorcinol method. The sum of the uronic acid released in the first and the second steps was referred to as the total polygalacturonate (total pectin) content. Reagent blanks, which contained all ingredients, but without additions of enzymes (PG and PME), were included in all steps of the reactions to eliminate any background from contaminating free uronic acid in the samples. The amount of the liberated methanol after PME digestion was also determined by the PME coupled enzyme assay method (above). Comparable data were obtained with both uronic acid and methanol measurements.

### Immunolocalisation of Methyl Esterified and De-esterified Pectin in Fibre Transverse Sections

Cotton fibres were fixed in 4% paraformaldehyde and 0.2% glutaraldehyde in 50 mM PIPES buffer, pH 7.2, at room temperature for 3 h. Following four washes in 50 mM PIPES buffer, samples were dehydrated in an ethanol series (25% to 100%), and embedded in LR White resin series (10% to 100%). Transverse sections of embedded fibres were cut at 2 µm thickness on a Leica UC6 microtome. Sections from the middle of the fibres were incubated with rat monoclonal JIM5 or JIM7 antibody (Plantprobes, UK), diluted 1∶50 in Phosphate Buffered Saline (PBS) overnight at 4°C. JIM5 cross-reacts with HG containing at least four contiguous unesterified GalA residues between two methyl-esterified residues, while JIM7 binds to regions with at least 4 adjacent methyl-esterified residues flanked by unesterified GalA [35]. Subsequently, sections were washed three times 10 min in PBS and incubated with Alexa Fluor 488 labelled goat anti-rat IgG (Sigma), diluted 1∶400 in PBS for 2 h at 37°C. After four 5 min washes in PBS, sections were mounted in 50% glycerol and were imaged on a Leica TCS SP2 confocal laser scanning microscope. Alexa Fluor 488 was visualized with the 488 nm channel of an Ar/ArKr laser.

## Results

### Genomic Diversity of Pectin Methylesterase Genes in Cotton

As PMEs are generally encoded by multigene families in most plants and only a partial fragment (Genbank Accession: ABH93358) of a single cotton PME (Carbohydrate Esterase Family 8) was present in the Carbohydrate Active enZyme (CAZy) database we sought to identify cotton PME orthologues, particularly those expressed in fibres, from amongst the extensive collection of cotton ESTs in Public databases. It should be noted that the two main cultivated species of cotton, *G. hirsutum* and *G. barbadense*, are both allotetraploids with an A- and a D-genome that are highly conserved both within and between the two species and also highly conserved between the tetraploids and their presumed extant diploid progenitors, *G. arboreum* (A_2_-genome) and *G. raimondii* (D_5_-genome). Homoeologous genes are often over 94% identical at the nucleotide level (eg., see [36]). The recent evolutionary origin of the tetraploids (1–2 mya) and their very narrow genetic base, therefore allows highly accurate gene assemblies to be generated from mixed ESTs from amongst the four different *Gossypium* species for which extensive ESTs have been lodged in GenBank. After blasting the translated cotton EST databases with PME protein sequences from *Arabidopsis* (AtPME2 and AtPME31, representing the two major types of plant PMEs, respectively [27]) and assembling those hits into contigs, we were able to identify at least 33 distinct expressed cotton PME genes from over 520 EST sequences derived from a variety of cotton tissues and species (sequences of the assembled contigs are given in Figure S1).

Subsequently, a draft assembly of the diploid *G. raimondii* genome was released [37] and this allowed a more extensive survey of *Gossypium* PMEs using the same search strategy. Although 102 gene models were identified in *G. raimondii* with a Blast hit to *AtPME2* or *AtPME31* and/or our 33 identified EST contigs, 21 of those were characterised as PME inhibitors (PMEI) by the NCBI Conserved Domain Database (CDD) Search tool and lacked a PME catalytic domain. This expanded the number of potential cotton PMEs from 33 to a total of 81 (Table S1). These PME genes were distributed across all 13 chromosomes of *G. raimondii* with several being clustered in groups of two, three or four genes in tandem order (Table S1) suggesting they have evolved through gene duplications and later divergence. The gene models encoded predicted PME proteins ranging from as little as 120 amino acids (aa) to one as large as 1262 aa, although generally they were less than 600 aa. The predicted PME inhibitor proteins were usually around 200 aa. Amongst the predicted PMEs, the smaller proteins generally only contained a single conserved PME catalytic domain when queried in the CDD, whereas the larger proteins contained a conserved N-terminal PMEI domain and a C-terminal PME catalytic domain.

**Figure 1:**
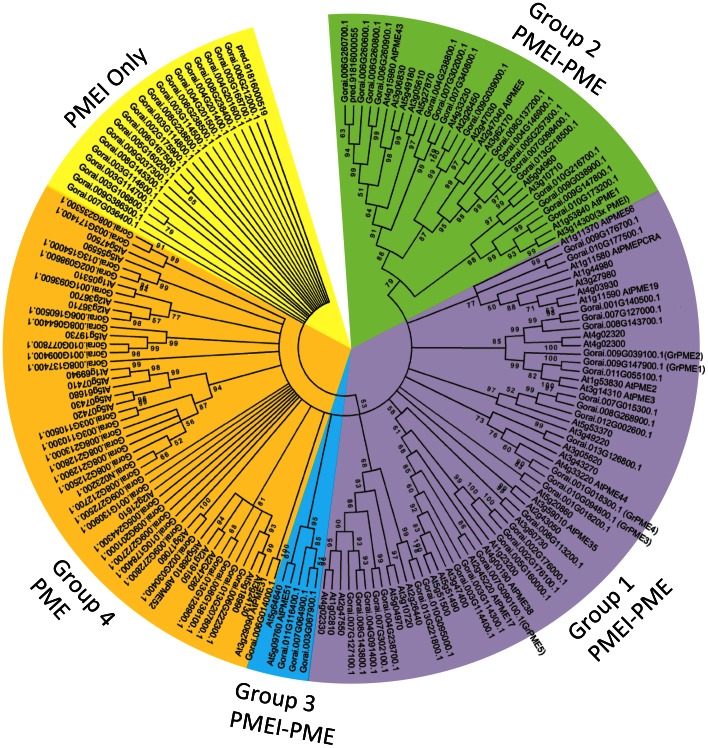
Phylogenetic Tree of Full-length PME and PMEI Proteins from *Gossypium raimondii* and *Arabidopsis thaliana*. The rooted tree was constructed using MEGA 5.0 with the Neighbor-Joining (NJ) method after alignment of the full-length amino acid sequences of 102 *G. raimondii* and 66 *Arabidopsis* putative PME or PMEI protein. Numbers at nodes indicate the percentage bootstrap scores and only bootstrap values higher than 50% from 1000 replicates are shown. Clades corresponding to the different PME groups identified by Louvet et al. (2006) are indicated in different colours. Gorai gene ID names are those of Paterson et al., (2013) (http://www.phytozome.net/cgi-bin/gbrowse/cotton_er/). The homologues of the five fibre-expressed PMEs are noted GrPME1–5.

A phylogenetic analysis of the encoded proteins of the 102 putative *G. raimondii* PME and PMEIs along with the 66 known and putative PMEs from *Arabidopsis* (Figure 1) clustered the cotton genes dispersed among the four different groups of plant PMEs previously defined in [28], with a separated clade that included all the PMEI only proteins. As they specifically bind to the active site of plant PMEs, PMEI proteins are thought to play important roles in modulating PME activity *in muro* during growth and development, but they have also been implicated in host defense responses to pathogens (reviewed in [8]). Their possible expression and functions in cotton fibres remains to be explored, so they are not considered further here. Relative to *Arabidopsis* the greatest expansion in PME genes appears to have been in the group 4 PMEs that contained over 64% more members than in *Arabidopsis*, whereas as the other three groups all had similar numbers in the two species (Table 1). Although most cotton PMEs have a related *Arabidopsis* protein they tend to group more closely with each other in small sub-clades (Figure 1) suggesting they may have specialised to new functions in cotton and we were particularly interested in those that might have a novel function in cotton seed fibres that are relatively unique plant seed epidermal trichomes not found in *Arabidopsis*.

**Table 1:** Expansion of PME Genes in Diploid *Gossypium raimondii* Compared to *Arabidopsis thaliana.*

PME Subgroup^a^	No. PME Genes in *G. raimondii*	No. PME Genes in *Arabidopsis*
1	29	31
2	18	14
3	3	2
4	31	19

a As defined by Louvet et al., (2006).

Based on the presence of matching ESTs in GenBank many of the cotton PME genes are expressed in young flower buds, developing ovules and in some cases cotton fibres (Table S1), although relatively few had abundant ESTs from cDNA libraries made exclusively from only fibres. Just five of the cotton PME genes (represented by Gorai.009G147900.1, Gorai.009G039100.1, Gorai.010G094800.1, Gorai.001G018300.1 and Gorai.007G090100.1 in *G. raimondii*), all PMEI-PME types, appeared to be expressed abundantly in isolated cotton fibres and these were studied in more detail in the two tetraploid cottons. Primers corresponding to the consensus sequences matching those genes were designed (Table S2) and the genes amplified from cotton fibre cDNA (both *G. barbadense* and *G. hirsutum*) and sequenced. The cloned cDNA sequences were extended by 5′RACE where required. The cloned cDNAs were highly similar to the corresponding genomic sequences from *G. raimondii* as well as the consensus nucleotide sequences assembled from Public ESTs (>99.3%), validating the use of the mixed *Gossypium* species ESTs in the assembly process to identify cotton genes. The cloned cotton genes were designated *GbPME1–GbPME5* (Genbank Accessions JX002994, JX002995, JX002996, JX002997, and JX002998) and *GhPME1*–*GhPME5* (Genbank Accessions JX002999, JX003000, JX003001, JX003002 and JX003003), each gene with a highly similar homoeologous partner (eg., *GhPME1-A* and *GhPME1-D*). The *GhPME1-5* genes were also highly similar to their matching orthologues in the other tetraploid, *G. barbadense* (>98% at the aa level). The deduced proteins varied from 514 to 582 amino acids and all clustered within the more abundant Group 1 PME proteins from *Arabidopsis* and other plants that have both a catalytic PME domain and a PME inhibitor (PMEI) domain similar to the pre-pro-protein domains of AtPME2 used as one of the query sequences.

**Figure 2:**
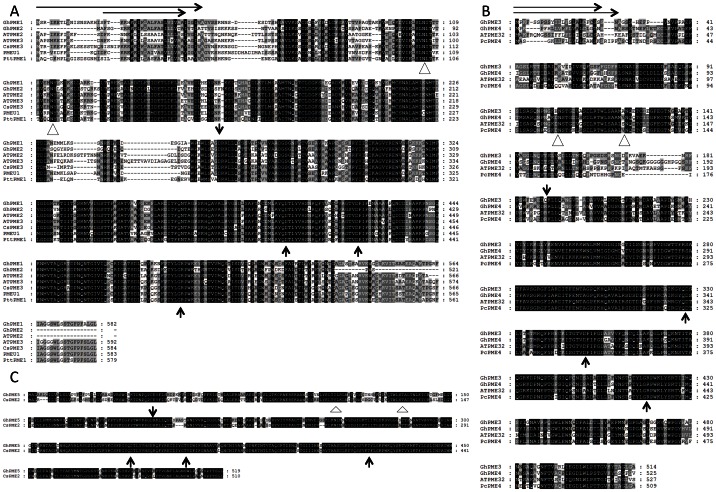
Alignment of Amino Acid Sequences of the Fibre Expressed Cotton PMEs with Other Closely Related Plant PMEs. All sequences are the full-length sequence where available aligned using ClustalW2 (http://www.ebi.ac.uk/Tools/clustalw2/index.html). Identical amino acids are in black, with less conserved residues in shades of grey. The catalytic residues Asp (D) and Arg (N) are indicated by vertical arrows below the sequences. The putative transmembrane domains are boxed. The signal peptide is shown by the horizontal arrows. The predicted cleavage sites for mature protein are indicated by vertical arrows above the sequences. The triangle shows the potential N-linked glycosylation sites. A) *G. hirsutum* PME1 and 2 aligned with PMEs from *Arabidopsis* (AtPME2, & AtPME3), orange (CsPME2 & 3), tobacco (PMEU1) and poplar (PttPME1). Note that the *GhPME1* cDNA is not full-length at the 5-end. B) *G. hirsutum* PME3 and 4 aligned with PMEs from *Arabidopsis* (AtPME32) and pear (PcPME4). C) *G. hirsutum* PME5 aligned with the PME from orange (CsPME2).

Cotton PME2-4 proteins were each predicted to have well defined signal peptides for targeting into the endomembrane system, while the cotton PME1 had a predicted signal anchor (uncleaved signal peptide), so all are expected to be deposited in the cell wall. As with other plant Group 1 PMEs, all five cotton proteins had an N-terminal PMEI domain, a putative processing motif (either RRLL or RKLL) followed by a C-terminal pectin methylesterase domain that is the mature protein present in the cell wall. The PMEI region may act as an auto-inhibitory domain and prevents untimely PME activity during transport, but is cleaved at or after the protein is secreted into the cell wall [27], [38]. Cotton PME1 and PME2 had predicted N-terminal transmembrane helical domains overlapping with their signal sequence or signal anchor (Figure 2A). There were no predicted transmembrane helices in cotton PME3, PME4 and PME5. The deduced protein sequences of cotton PME1 and PME2 share 67% identity, while PME3 and PME4 shared 78% identity and group together in the phylogeny (Table 2, Figure 1). Cotton PME5 was different from the other PMEs with sequence identities between 40–50% (Table 2), consistent with its derived phylogeny (Figure 1). The positions of the catalytically significant aspartate and arginine residues of the pectinesterase active site [39], [40], [41], [42] were conserved across all five cotton PME proteins (Figure 2), as were a number of other functionally significant residues in pectin binding, so they are all expected to be functionally active as pectin methylesterases. Two potential N-linked glycosylation sites, specified by the sequence Asn-X-Thr/Thr, were found in their N-terminal regions (Figure 2) as has been observed in other PMEs [21]. Alignment of the amino acids of cotton pre-pro-proteins showed considerable identity at the C-terminal PME end whereas the amino acid sequences at the N-terminal signal peptide and PMEI end were more variable (Figure 2). This has been noted in the alignments of other plant PME protein sequences [43]. Based on the alignments and homology predictions from the Conserved Domain Search (NCBI), GhPME1, GhPME2, GhPME3, GhPME4 and GhPME5 had the predicted mature proteins of 28 kD with pI 9.2, 24 kD with pI 7.09, 33 kD with pI of 9.09, 34 kD with pI of 8.57 and 28 kD with pI of 9.09, respectively.

**Table 2:** Percentage Amino Acid Sequence Identities of PMEs from Cotton and Selected Other Plants.

	GhPME1	GhPME2	GhPME3	GhPME4	GhPME5
GhPME1	–				
GhPME2	**67**	–			
GhPME3	41	37	–		
GhPME4	42	36	**78**	–	
GhPME5	50	40	44	44	–
AtPME2	**69**	**64**	41	40	41
AtPME3	**72**	**64**	41	40	43
AtPME32	42	37	**65**	**63**	44
PcPME4	44	39	**70**	**70**	46
CsPME2	47	41	43	43	**76**
CsPME3	**79**	**66**	41	40	45
PMEU1	**76**	**66**	39	40	45
PttPME1	**74**	**64**	40	40	45

Proteins Over 50% Identical are Indicated in Bold Type.

Note: AtPME2, 3 & 32 (Accession Nos. NP_175786, NP_188048 and Q9LXK7) from Arabidopsis, PcPME4 (Accession no. BAF42041) from pear (*Pyrus communis*), CsPME 2 & 3 (Accession no. O04887 & P83948) from orange (*Citrus sinensis*), PMEU1 (Accession no. Q43143) from tobacco, PttPME1 (Accession no. CAC01624) from *Populus tremula* x *tremuloides.*

### Stage-specific Expression of *PME* Genes during Cotton Fibre Development

**Figure 3:**
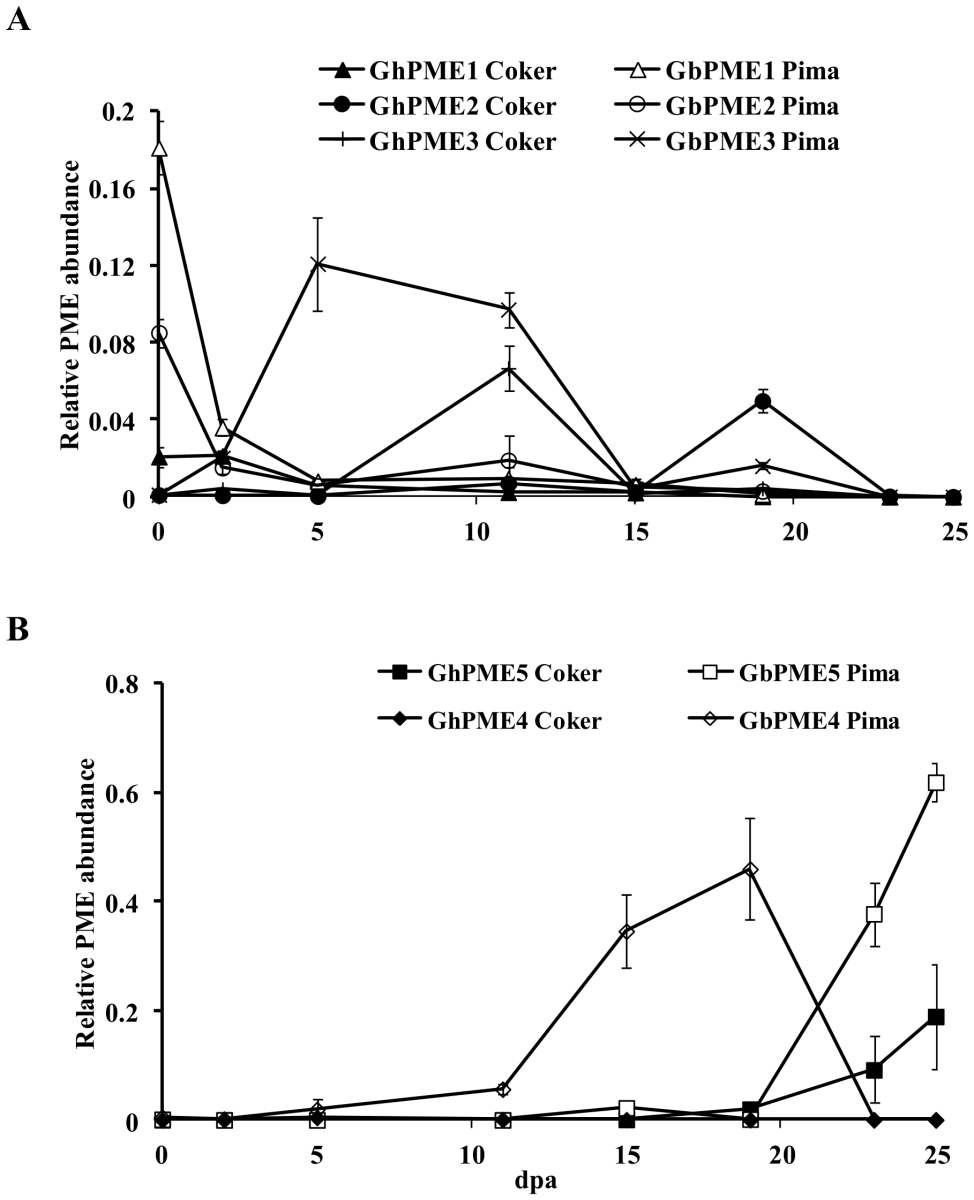
Expression Levels of Fibre-PME Genes throughout Fibre Development in Two Species of Cotton. Expression was measured by quantitative real-time PCR on cDNA from whole ovules for 0, 2 and 5 dpa and from isolated fibres thereafter from either *G. hirsutum* (Coker 315) or *G. barbadense* (Pima S7). The data were normalized using a reference ubiquitin gene (EU604080). Error bars indicate standard errors (n = 6, two biological replicates each with three technical replicates). dpa, days post anthesis.

Transcript levels of the five cotton fibre-*PME* genes in ovules and fibres were determined by quantitative real time PCR. The gene-specific primers used detected both homoeologues of each gene within each species and the orthologous genes in both *G. hirsutum* and *G. barbadense* as was confirmed by sequencing the PCR products (not shown). The primers amplified the genes from the two species with similar efficiencies, so their relative expression levels in the two species are directly comparable. Expression of each of *PME1*–*PME5* gene was fibre development stage-specific, particularly in Pima S7 cotton (*Gb*) where the genes were all generally more highly expressed (Figure 3). The mRNA levels of *PME1* in Pima S7 fibres were higher at 0 dpa, and decreased by about 22 fold at 5 dpa, then remained at very low levels for the rest of fibre development (Figure 3A). Because of the difficulty in removing fibres from young ovules, the 0, 2 and 5 dpa samples were from whole ovules so this expression may not be attributed just to fibre initials and young elongating fibres and could also represent expression in the integument, or developing zygote. The transcripts of *PME1* in Coker 315 (*Gh*) were 9 fold lower than those in Pima S7 fibres at 0 dpa, then declined to very low levels over the rest of fibre development. *PME2* in Pima S7 fibres had a similar expression pattern to *PME1*, reaching a maximum at 0 dpa, but was about 2 fold lower than *PME1*. *PME2* expression in Coker 315 fibres remained low during the whole of fibre development, except for a transient increase at 19 dpa. The mRNA levels of *PME3* in Pima S7 fibres increased at 5 dpa and remained at similar levels till 11 dpa, then decreased by about 22.5 fold at 15 dpa (Figure 3A). *PME3* in Coker 315 fibres was un-detectable before 5 dpa, but increased dramatically to a high level at 11 dpa then decreased by 17 fold at 15 dpa, but was always lower in expression than in Pima S7 fibre. *PME4* mRNA in Pima S7 fibres increased substantially by 11 dpa and peaked at 19 dpa, then dramatically decreased by 23 dpa (Figure 3B). The mRNA levels of *PME4* in Coker 315 fibres remained low at all stages during fibre development (Figure 3B). *PME5* mRNA was undetectable before 20 dpa in both Pima S7 and Coker 315 fibres (Figure 3B), but increased thereafter. Again, mRNA levels of *PME5* were 3.2 fold higher in Pima S7 than in Coker 315 fibres by 25 dpa and were more abundant than any of the other cotton fibre *PME* genes. Overall, the most highly expressed *PME* genes in Pima S7 fibres were *PME4* and *PME5*, but these appeared to be at different stages of fibre development, with *PME4* being most abundant during late elongation and the transition to SCW thickening while *PME5* was most abundant during SCW thickening.

### Temporal Changes in Total PME Enzyme Activity during Fibre Development in Two Cotton Species

**Figure 4:**
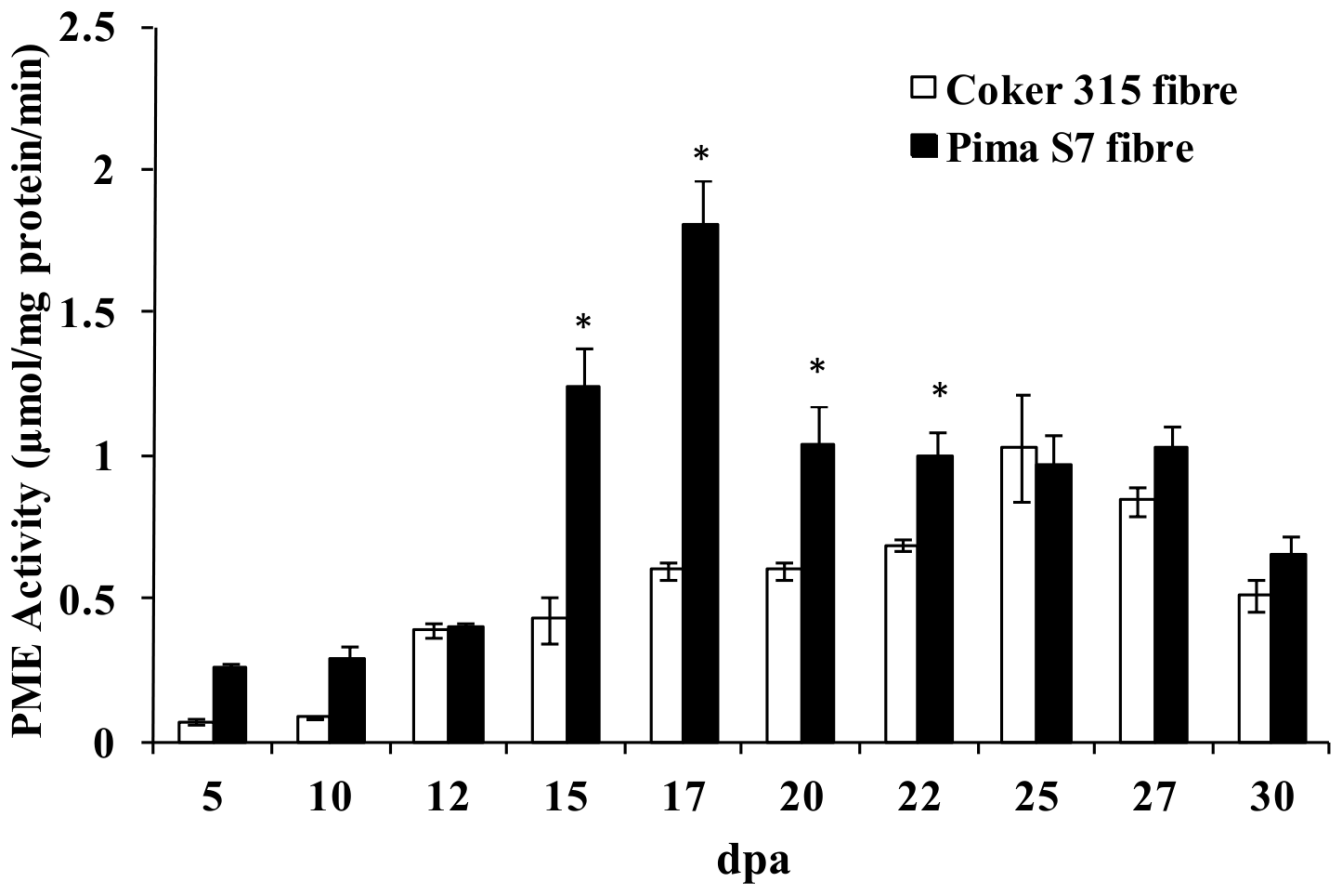
PME Enzyme Activity in Two Cotton Species Determined throughout Fibre Development. Soluble PME enzyme activity was measured in isolated fibres from developing seeds of *G. hirsutum* cultivar Coker 315 and *G. barbadense* cultivar Pima S7 using citrus peel pectin as a substrate in a coupled enzyme reaction as described in Materials and Methods. Error bars indicate standard errors (n = 9, three biological replicates each with three technical replicates). dpa, days post anthesis. * indicate values for *G. barbadense* that were statistically different to those in *G. hirsutum* by a t-test (P<0.05).

Although it was not possible to measure the activities of each of the different PME isoforms separately, total PME activity should reflect the major biochemical processes in the cells at the different stages of fibre growth. Total PME enzyme activity in Pima S7 fibres was low before 15 dpa, increased rapidly between 15 and 17 dpa and then declined to a moderate, but stable, level up till 30 dpa (Figure 4). PME activity in Coker 315 fibres was low at the start of fibre development and increased gradually to peak at 25 dpa, declining slightly thereafter. Total PME activity from 25 dpa was of similar magnitude in the fibres of both species. Consistent with the higher transcript abundance, the overall PME activity during elongation in Pima S7 fibres was substantially higher (about 3 fold) than in Coker 315 fibres, particularly between 15 and 17 dpa when there was a burst of PME activity (Figure 4).

### Extractable Pectin Content Changes in Fibre Cell Walls during Fibre Development in Two Cotton Species

The temporal changes in *PME* gene expression and total PME enzyme activity would be expected to impact on the content and properties of the pectin in the fibre cell wall and we sought to confirm this by chemical and biochemical analysis. Although HPLC and FTIR methods that are fast and sensitive have been developed for the quantification of pectin [44], [45], they require access to specialised equipment not required for more simple colorimetric assays. Many of these simple chemical methods, however, utilize strong acids with the potential for non-specific measurement of other sugars. We adapted an enzymatic method for the determination of polygalacturonates extracted from plant tissues. It uses commercially available PME to remove methylesters and polygalacturonase to hydrolyse polygalacturonates that were then assayed using a colorimetric reaction with naphthoresorcinol reagent. Interference by other sugars present in the tissues is thereby minimized. Traditional colorimetric assays (eg., [46]) and the new enzymatic method generated comparable results for commercially available pectins (Figure S2). In comparison to the straight colorimetric methods this protocol can also be adapted to assay both esterified and de-esterified pectin separately.

**Figure 5:**
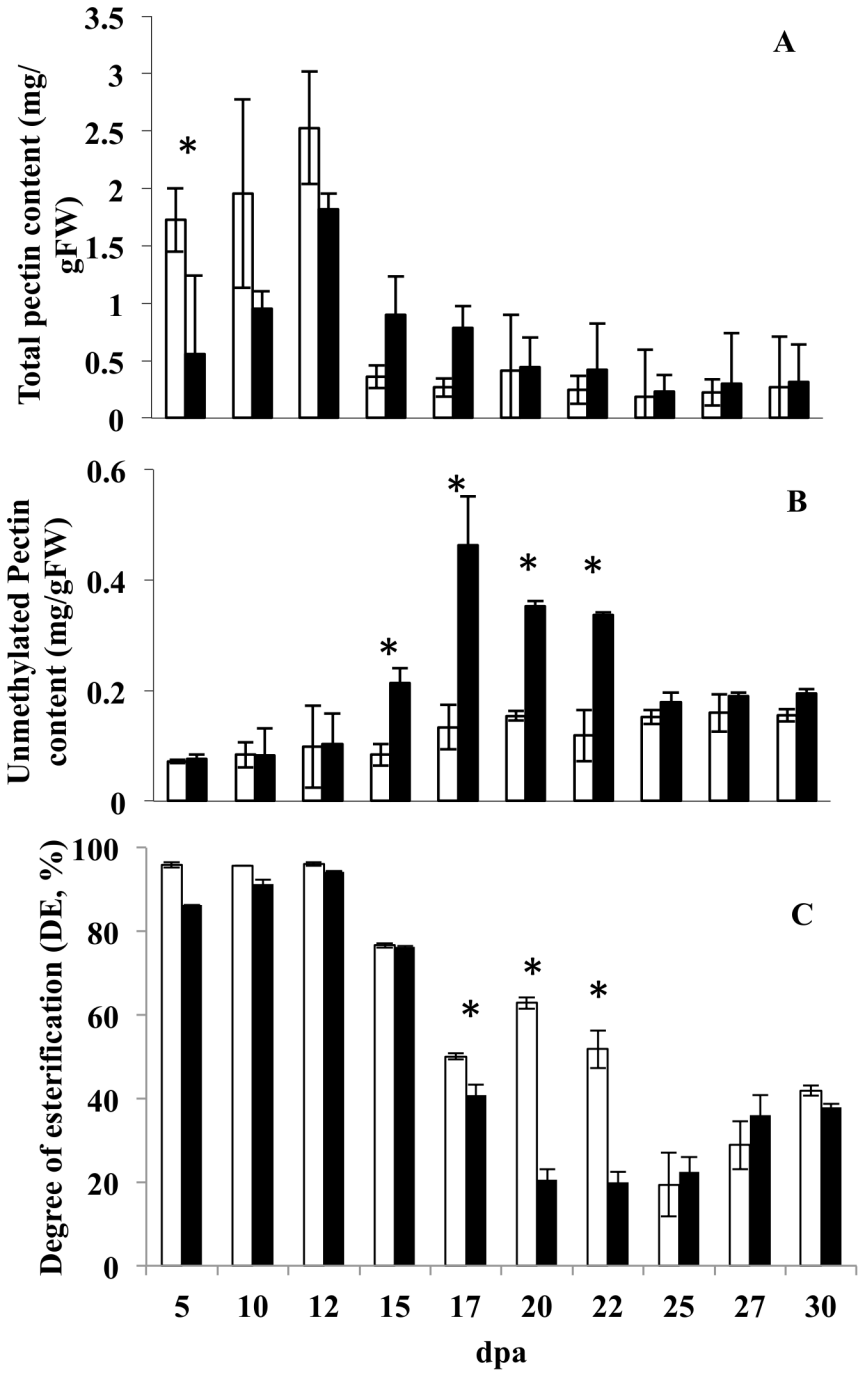
Fibre Cell Wall Pectin Content and Composition Changes during Fibre Development in Two Cotton Species. Isolated fibres from *G. hirsutum* cv Coker 315 (open bars) and *G. barbadense* cv. Pima S7 (solid bars) were extracted and (A) total pectin as uronic acid; (B) unmethylated pectin and (C) percentage of unmethylated pectin in the total pectin, determined as described in Materials and Methods. Error bars are standard errors from two biological replicates and three technical replicates. dpa, days post anthesis. * indicate values for *G. barbadense* that were statistically different to those in *G. hirsutum* by a t-test (p<0.05).

The concentration of extractable pectin (containing both methylesterified and de-methylesterified polygalacturonic acid) was high in early stages of fibre development and increased to around 2 mg/g Fresh Weight (FW) by 12 dpa in both species (Figure 5A). Pectin content of the Coker 315 fibres was significantly higher than that in the Pima S7 fibres, but only at 5 dpa on a fresh weight basis. There was a sharp decrease in pectin concentration at 15 dpa. The decrease was more pronounced in Coker 315 than in Pima S7 fibres, resulting in more than a two fold higher pectin concentrations at 15 dpa and 17 dpa in Pima S7 than in Coker 315 fibre cell wall extracts (Figure 5). Pectin concentrations continued to decrease in both species reaching 0.4 mg/gFW by 30 dpa in both Pima S7 and Coker 315 fibres (Figure 5A).

### Structural Remodelling of Pectin in Fibre Cell Walls during Fibre Development Differs in Two Different Cotton Species

The same assay, but without prior PME digestion, was used to measure the amount of de-esterified pectin in the cell walls of the fibres from both species. Although total pectin concentrations were high, the amount of de-esterified pectin was low at the early stages of fibre development before 12 dpa in both Pima S7 and Coker 315 fibres (Figure 5B). More than 90% of the extractable pectin was methylesterified before 12 dpa (Figure 5C), consistent with previous reports of the low level of de-esterification in newly synthesised pectin. Concentrations of de-esterified pectin started to increase at 15 dpa in Pima S7 fibres and reached a peak at 17 dpa (Figure 5B), coinciding with the high PME enzyme production at this stage. About 60% of the extractable cell wall pectin was in the de-esterified form and this increased to 80% over the next few days, only dropping slightly by 27 dpa. Total concentrations of de-esterified pectin started to decrease from 20 dpa in Pima S7, but stabilised from 25 dpa onwards. The concentration of de-esterified pectin in Coker 315 fibres, on the other hand, started as low as in Pima S7 fibres and only increased gradually throughout fibre development, reaching a similar level to that in Pima S7 fibres by 25 dpa. The DE still dropped to 50% by 17 dpa (Figure 5C) remaining at that level for the next 5–6 days and then dropped to around 20% by 25 dpa in Coker 315 and by 20 dpa in Pima S7. The DE of the extractable pectin in the two species was therefore reasonably similar both early (high) in fibre elongation (before 12 dpa) and later (low) during cell wall thickening (after 25 dpa), but they were quite different during the critical period from 17–22 dpa when the fibre is transitioning from elongation to SCW production (Figure 5C).

### Fluorescent Immunolabelling of Esterified and De-esterified Pectins in Fibre Cell Walls

**Figure 6:**
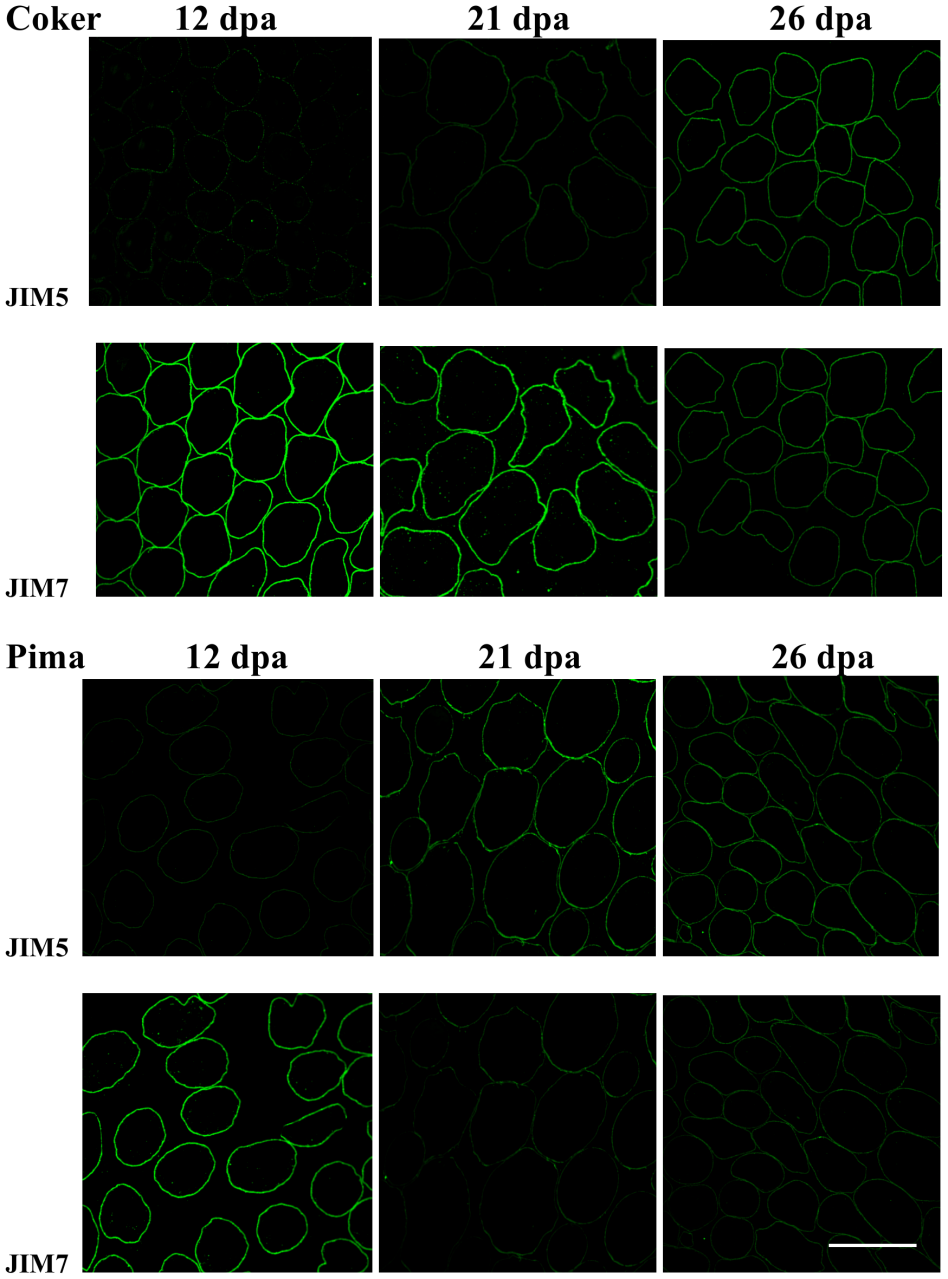
Localization of Pectin Epitopes in Cell Walls of Cotton Fibre Transverse Sections at Different Stages of Fibre Development. Immunolocalization of low-methyl-ester pectin (JIM5 epitope; top panel in each section) and high-methyl-ester pectin (JIM7 epitope; bottom panel in each section) in fibre cross-sections of two cotton species. Top two panels are *G. hirsutum* cultivar Coker 315 and the bottom two panels are *G. barbadense* cultivar Pima S7. Sections were taken from the middle of the fibres at 12, 21 and 26 days post anthesis (dpa). Scale bar = 50 µm for all images.

Two anti-pectin antibodies (JIM5 and JIM7) that distinguish between different degrees of methylesterification were used to investigate the DE of the pectin in cross sections of fibres at different stages in their development. At 12 dpa, JIM5 labelling was weak in both Pima S7 and Coker 315 fibres, whereas JIM7 labelling was very strong (Figure 6), indicating that the methylesterified pectin content was high at this stage of peak fibre elongation, consistent with the enzymatic determinations (Figure 5A). At 21 dpa, however, JIM5 labelling had increased substantially, especially in Pima S7 fibres and this was accompanied by a corresponding decrease in JIM7 labelling of the fibres in this species. The increase in JIM5 labelling was not as obvious in Coker 315 fibres at 21 dpa (Figure 6) and JIM7 labelling had remained high. At 26 dpa, JIM7 labelling had decreased markedly relative to 12 dpa, while JIM5 labelling was high both in Pima S7 and Coker 315 fibres, again consistent with the biochemical analyses (Figure 6). These data indicate that the primary wall of fibres were enriched in HG with a high DE during rapid elongation, but this was remodelled *in muro* (presumably by PME) to have a lower DE by the end of the elongation phase and into the secondary cell wall thickening stage. This remodelling occurs earlier in *Gb* than *Gh* fibres.

## Discussion

Pectin is a major component of the primary cell wall and middle lamella of dicot plants and undergoes complex remodelling that plays an important role in regulating cell wall expansion, elongation and adhesion [2]. However, little was previously known about pectin structure and its subsequent remodelling by PMEs in cotton fibres during their development. In this study, we have shown that, like other plants, there are a very large number of PME genes in cotton and these are likely to have a variety of different functions in different tissues and at different times. Among the fibre-expressed PME genes we studied in detail there were also multiple forms, each with substantial absolute and temporal differences in transcript abundance during fibre development that together resulted in temporal differences in total PME enzyme activity and alterations in the extent of de-methylesterification of cell wall pectin in fibres. Their differences in protein sequence and domain structure and the timing of their expression during fibre development suggest that they may all have quite different functional roles in the fibre.

Consistent with its important matrix function in PCW biogenesis, total extractable pectin in *Gb* and *Gh* fibres was shown to be highest during rapid elongation when new PCW is being synthesized. It declined significantly (as a proportion of fibre fresh weight) as the fibres switched their metabolism from primary to secondary cell wall synthesis when the major mass of the fibre becomes crystalline cellulose. The pectin from the rapidly elongating fibres was shown to be largely methylesterified and so would be more elastic to allow for cell wall expansion driven by the high turgor pressure in the fibres [47]. Over time the pectin was significantly remodelled and became largely de-esterified and this correlated with the rising levels of total PME enzyme activity. Highly de-esterified pectin is expected to form more rigid gels and provide resistance to further fibre cell wall expansion. By the time of peak SCW deposition at around 26 dpa pectin DE in the fibre walls was very low and stayed largely stable thereafter while PME enzyme activity continued to remain high, long after fibre elongation had ceased.

Pectin remodelling is clearly a critical process in regulating cell wall expansion in pollen tubes, elongating stems or hypocotyls and wood fibre elongation as well as fruit ripening in a number of plants (reviewed in [48]), and there is now accumulating evidence that pectin amount and DE might also be critical in regulating different aspects of fibre cell expansion and elongation. Pang and colleagues [49], for example, have reported that pectin synthesis genes are up-regulated in 10 dpa cotton ovules relative to a fibreless mutant and that exogenous nucleotide sugars that are precursors to pectin (UDP-Rhamnose, UDP-Galacturonic Acid and UDP-Glucuronic acid, but not UDP-xylose or the free sugars) can enhance fibre elongation in cultured cotton ovules, although in this case they did not examine any differences in the methylesterification of the pectin in those fibres. Young cotton fibre cell walls have also been observed to contain an outer sheath enriched in de-esterified pectin while the adjacent epidermal cells do not have this structure [50]. Singh and colleagues [51] have suggested that this may be the precursor to a pectin-rich middle lamella-like structure that they observed surrounding groups of fibres during the fibre elongation period that bound them together in a tissue-like bundle that was degraded at the end of elongation to release the individual fibres. The pectin in the middle lamella, unlike the fibre PCWs, had a low DE as it was labelled with JIM5 antibodies.

There was a relatively low level of PME activity during fibre elongation and this correlated well with the high level of methylesterification observed biochemically and immunologically during this time. Most of the PME activity in fibres was present later during the transition to SCW production or even later still when the fibre walls would not need to be as extensible. Some PME isoforms are, however, expressed during fibre elongation and so must have a different function to those expressed later in development. They may either be acting randomly on the pectin chains (there are few blocks of de-esterified residues able to bind the JIM5 antibody by 12 dpa) to assist elongation through their effects on cell wall pH or acting more specifically on the middle lamella layer reported in [51]. As the middle lamella pectin has been suggested to be de-esterified it must be modified *in situ* by specialised PME enzymes shortly after synthesis and these would need to be produced from quite early on in development (possibly one or more of PME1-3, that are all expressed mostly before 15 dpa). How these enzymes would be partitioned away from the pectin in the fibre walls that has a high DE is unknown. Our data would suggest that the middle lamella, if highly de-esterified, must represent a very small proportion of the total extractable pectin, since the bulk of the pectin present during fibre elongation was still methylesterified (Figure 5), consistent with the earlier models of turgor driven elongation of a highly extensible cell wall during the first 12–15 days of fibre growth [47]. The amount of de-esterified pectin extracted from fibres increased sharply after about 17 dpa in both species of cotton. As *PME4* in Pima S7 was the most abundant PME isoform at this stage it is likely to have this role in remodelling the cell wall pectin to change its extensibility properties that along with SCW formation might be expected to slow down the rate of fibre elongation.

*PME5* was elevated in expression after *PME4* had declined and appears to be specific to the SCW stage when the fibres have largely ceased to expand or elongate. *PME5* is the same gene reported in [51] as possibly being involved in priming the degradation of the middle lamella that forms between younger fibres, but it would appear to be too late in expression to have this as a major role. What could *PME5* be doing during this wall thickening stage when total PME enzyme activity is still quite high, and most of the extractable pectin is already de-esterified? PME isoforms have been identified in poplar that are expressed during xylogenesis in wood forming tissues and have been suggested to have a role in regulating lignification mediated through the binding of peroxidises to Ca^2+^-pectate [52]. Cotton fibres contain no lignin [53], but express many of the genes for the production of the phenylpropanoid precursors of lignin [36] so the two dominant cotton PME isoforms PME4 and 5 that are expressed during SCW production may have a similar role in stiffening the fibre PCW and/or strengthening the cellulose microfibrils in the secondary wall by promoting peroxidase mediated cross-linking to phenylpropanoid compounds like ferulic acid, rather than polymerisation of monolignols into lignin. The precise role of these two major fibre PME isoforms, however, will need to be determined in cotton plants where their activity has been manipulated by silencing or over-expression.

*PMEs* belong to large multigene families in most plant species [27], [54] with 66 known in *Arabidopsis* [48]. The growing availability of extensive genomic resources for different cotton species, and the release of a full genome assembly for one of the sub-genomes in cultivated cotton have allowed us to determine that cotton also has a very diverse complement of *PMEs*, many more than in *Arabidopsis*, so they must have evolved new or more specialised functions in this genus. Many of the *Arabidopsis* genes have been shown to be either very ubiquitous with a general role in plant growth or conversely to have quite low expression or at least very narrow tissue- or cell-specific expression [48] and cotton species are likely to be similar with their 81 or more different PME types. Plant *PME* genes have been classified as either type I or type II [27] depending on the presence or absence of particular protein domains. Type I PME genes (designated Groups 1–3 in the phylogenetic analysis of Pelloux et al., [48]) contain a pro-region with a conserved PME inhibitor (PMEI) domain as well as the pectin methylesterase catalytic domain, have two or three exons and are usually highly expressed in multiple tissues compared to the more restricted expression of the Group 2 and 4 type genes [48]. Type II PME (Group 4) genes have a signal peptide for secretion, but don’t have a PMEI region and typically have five exons, and are more similar to *PME* genes of phytopathogenic bacteria and fungi [27]. Cotton appears to have had an expansion in these type II PMEs, often in small clades distinct from their closest *Arabidopsis* orthologues, but none of these Type II PMEs appear to be expressed at high levels in cotton fibres, so they must have roles in other tissues or cell types (in *Arabidopsis*, many Group 4 PMEs are expressed in the pollen where they have roles in tetrad separation and pollen tube growth [15] or in some cases in the young silique within the developing seed [28], [48]). Both types, however, have been found as their smaller processed mature PME form in cell walls of other species, so may still be functionally redundant. The five cotton PME genes we identified were all Type I PMEs and had the characteristic signal peptide (or signal anchor), PMEI domain and pectinesterase domains of the type I pre-pro-PMEs [27] The pro-region appears to have roles in both extracellular targeting and inhibitory effects on PME enzyme activity while it is being transported to the cell wall [15]. The processing steps of pre-pro-PME into pro-PME and eventually into the functionally mature PME in the cell wall have all been shown to be potential levels of regulation of PME activity [27], [55]. In our phylogenetic analysis, the fibre-expressed PMEs were interspersed among the *Arabidopsis* Group 1 PMEs and all have a reasonably close *Arabidopsis* orthologue that is relatively broadly expressed [48], but as *Arabidopsis* does not have seed fibres the cotton genes must have diversified to this additional function in seed epidermal hair development.

As the two cotton species we studied vary quite markedly in fibre quality we were interested in any differences in the timing or extent of cell wall remodelling in the respective species, as this may provide clues as to why *Gb* has the much longer and finer fibres that are commercially more valuable. Earlier transcriptome comparisons between *Gb* and *Gh* fibres [36] had already highlighted significant differences in both pectin synthesis and pectin modification gene expression between these two species, particularly during the rapid elongation phase. Pectin synthesis genes (eg., UDP-glucose 6-dehydrogenase and UDP glucuronate 4-epimerase) were more highly expressed in *Gh* at 7 dpa than in *Gb* and this accords with the higher total pectin content of *Gh* fibres reported herein. At least one PME gene (*PME4* or a close homologue), on the other hand, was reported to be more highly expressed in 7 and 11 dpa in the fibres of Pima S7 than in a *Gh* cultivar Siokra 1–4 and its expression at 10 dpa was positively correlated with higher final micronaire (a measure of fibre fineness and maturity) amongst a group of inter-specific cotton RILs. We also observed a higher expression of *PME4* in Pima S7 than in Coker 315 between 15 and 19 dpa, the period at the end of elongation when the fibre is transitioning to SCW production.

Although they differed slightly in the total amount of extractable pectin, both species had extractable pectin of similar DE during the rapid fibre elongation stage (high DE) and later during SCW thickening (low DE), but surprisingly they were most different during the transition between these two stages, with the *Gb* fibres extracts becoming less methylesterified earlier than *Gh* because of a burst in PME enzyme activity. Although still contentious, cotton fibre elongation is thought to proceed by a combination of mainly intercalary or diffuse growth and some tip growth (reviewed in [56]) all driven by the high turgor pressure in the fibre cell during the elongation phase [47]. While perhaps counter-intuitive, a higher proportion of de-esterified pectin in the *Gb* fibres, especially away from the tip, during late elongation would be expected to make the walls stiffer, constraining any further radial expansion (and so keeping the *Gb* fibres finer than those in *Gh*) while enhancing further turgor driven longitudinal elongation from the tip (making their fibres longer). We have measured very similar rates of elongation in *Gh* and *Gb* fibres and similar timing for the onset of SCW cellulose deposition under the growth conditions we used for this experiment [36]. Avci and colleagues [57] also reported very similar rates of early fibre elongation and onset of SCW synthesis in a different pair of Gh and Gb cultivars that also had very different final fibre length, strength and fineness and correlated that with differences in cell wall matrix xyloglucan polymers and cell wall loosening enzymes like xyloglucan endo-hydrolases (XEH) that were much more highly expressed in Gb fibres. So it is likely that the balance between radial and longitudinal expansion controlled through cell wall polysaccharide remodelling enzymes like PMEs and XEHs that modify the matrix structure and composition *in muro*, rather than the timing of the transition to SCW production that are the keys to the different final length and fineness of the fibres between these species. Although these changes may only be occurring over a few days there only has to be a few millimetres difference in final fibre length, for example, to be a commercially significant difference in fibre quality.

### Conclusion

Cotton has been shown to contain a diverse array of PME genes, many expressed ubiquitously, but many were also expressed highly in flowers and ovaries. Only a few specific PME isoforms were expressed in the specialised seed hairs or fibres of cotton and each of these showed a distinct temporal pattern of expression suggesting they may have separate functions in the remodelling of the pectic matrix at different times during fibre development. Some of the genes were expressed throughout rapid fibre elongation and so may be contributing to cell wall loosening by lowering wall pH and assisting turgor driven wall extension, or acting on a pectin containing middle lamella-like structure recently reported to surround groups of fibres during elongation. Overall, however, the level of PME enzyme activity during fibre elongation was quite low. The two most abundantly expressed genes were transcribed later in fibre development and correlated well with the total amount of enzyme activity and higher levels of de-esterified pectin observed at those later stages. It is suggested that these forms of PME were responsible for the observed remodelling of the pectin near the end of fibre elongation and may be contributing to wall stiffening. Why levels of PME remain high during subsequent SCW formation when fibre elongation is minimal remains unclear. The timing of the remodelling of the cell wall pectin was very different in a *Gb* species of cotton with longer and finer fibres than *Gh* cotton and this and other change to wall composition and extensibility is proposed to be physically constraining radial expansion while promoting further longitudinal expansion of those *Gb* fibres. Targeted alteration of *PME* gene expression and PME enzyme activity could therefore be a key to improving cotton fibre quality in *Gh* varieties either through selection in breeding or GM approaches.

## Supporting Information

Figure S1 Consensus sequences of 33 distinct cotton PMEs assembled from EST sequences from *G. hirsutum*, *G. barbadense*, *G. arboreum* and *G. raimondii* available from GenBank, January 2012.
(DOCX)

Figure S2 Comparison between colorimetric and enzymatic methods for quantification of total extractable pectin in cotton fibre cell walls. Comparable results of estimated total pectin extracted from *G. hirsutum* fibre analysed either by the new enzymatic method described in Materials and Methods and the traditional colorimetric assay of Filisetti-Cozzi and Carpita [46]
(DOCX)

Table S1 Genomic diversity of PME genes from a diploid cotton (*Gossypium raimondii*) with a fully sequenced genome.
(XLSX)

Table S2 PCR primers used for the cloning of the cotton PME cDNAs and for quantification of expression levels by quantitative realtime PCR.
(DOCX)
