# Supplementary material for: Pectin Methylesterase and Pectin Remodelling Differ in the Fibre Walls of Two Gossypium Species with Very Different Fibre Properties
Source: PLoS One. 2013 Jun 5;8(6):e65131. doi: 10.1371/journal.pone.0065131 (PMC3673955; doi:10.1371/journal.pone.0065131)
Supplement: Figure S1 — Consensus sequences of 33 distinct cotton PMEs assembled from EST sequences from G. hirsutum, G. barbadense, G. arboreum and G. raimondii available from GenBank, January 2012. (DOCX) [file pone.0065131.s001.docx]

**Figure S1.** Consensus sequences of 33 distinct cotton PMEs assembled from EST sequences from *G. hirsutum*, *G. barbadense*, *G. arboreum* and *G. raimondii* available from GenBank, January 2012.

>PMECon1

ggaaaattttatgattttttgagggtttgttaagaataaatagaaaacaatggcgaacaatgttgctgttattggtatctgttcggtgttcttggtggcagcagtggtggcagcagtggtgggtgttacgcatttaaaaaacaacggtgattcgaaaagcggtgaaatatcgagttcgacgaaagcggtgcaaactgtttgtcaacccacacagttcaaagaggcatgcgagaagagcctggagtcatctaattccaccgaccctaaggagctgataaggacgagtttccaagcagcgatagaggaaataaggaaagtgttggcgaattcagcgacgatacaagacttgaataaagatgacaacaacaaagcagcccttaaggtttgtaaagaagtattggatttatcaattgctgacctgcaactttcatttgataagctggggggatacgagatgagtaagatcggtgattacttactaaacctgagggtgtggttaagcggtgccttaactaccctacaaacatgtgtagactcatacgcggaggtaaataaagaacaagcggaaaagatgaattcaattttgaagacttcaatggagcttacccaaaattctttaaccatggtgactaagttatccaccatcttgaatgaccttaacattccaggcatcaatatcgacacaactgggtttgaacgtaagctcctgtctaacgatggacctgagtggatgggccacgctgagaggagactacttcaagcaaaaccaacagatttgaagcctaacgttgtggttgctaaggatggtagtgggaaatatgataccatcaccaaggccttggctgaagtccctccaaaaagccccaatcgatttattattcacattaaggctggtacttacaaggaacaagtccttttacctaaggagatgactaatgttatgttcatcggtgatggtccaactaagaccatcatcaccaatgaccttaactgcattagggatcatccgcttaaaacattcggcactgcaacggttggtgtggatggggctggtttcatggccagggacattggattcgagaacacagcaggacctcctggtcatcaagcagtagcttttagggcaacatgtgatatggtcatcatgttcaattgtcatttcaatggataccaagacaccctttatgctcacaaacagaaacaattttatagggattgtctcatcagcgggacagtggatttcattttcggggatgcggcgagtgtgttccaaaattgtctgatcattgcgaggaagcccggtgaaaaccaaaacaacatgattacagcccacggaaggaaattcccatacactcactcagccattgtgcttcaaaattgtaccatctcaggcgcccctgactacctcccggtgaaggacaagagcaagacgtaccttggtcgtccttggaaagctttggcgaggaccatcataatgcaaagcaatattgatgacatcattacaccagagggttactcccccatggaaggcaccgtaggactagacaccggttactttgtagagttccaaaataggggaccaggggctaaaaccgaaggcagggtcaaatggccagctatcaagacaatcgatataaatgaagccaagaaatggactcctggcgtctttcttgaaactgaaactgacaaatggatttcgcaaaccggcactccttgttatcctgacatggtccctggagtgtaaacttccaaaaccatcgccattagtattcttttattatcattattagtaatcatgttgtcgattattgattaagttaaacaatttttttcctatgttttatttatattaataatatattctttattatgg

>PMECon3

tccaaaactgtcagatcgtcgtgaggaagccaggtgagaaccaccccaacatggttacagcccccggaaagaaagaagaagaaactaccccggcttttgtgcttcaaaattgtaccatttggggggcccctgactccctcccggtgaaggacaagaacaagaggtcccttggttgtccctggaaacagtttgggacaactgtcataatgcaatgtcaaatggatgacatcattcccccagggggttactcccccatggaaggccccgtaggattggcccctggttattttgccgggttccaaaataggggaccaggtgccaaccccgggggcagggtcccatggaaagctttcaagaaaatcgatatggatgaagccatgaaatggactcctcgtgtttttttaaaatccgaggaatggggggcacaaacgggggttccctttgatcctgatatgcccccgggggtttaaactttcaaacccatcgccattagtttttttttattatcattatttgaaaaaatgttgttgattattgattaatttgaacaactttatcctatttttataataataatatattcaactttttttggggcc

>PMECon4

aaagccattagacctgtctgatctctcgcggcgcctataaaataaacaaatatcttttcaccttctccggcatcacctttcaatggcttccaccatttccaaactctctatcatcctccatttcaccctctttttatcttttgctttagctctgaactctatttcaactactcccctaagctctaatctctcttccatacgaaatttttgccaatcaactccatatccagatgcttgtttcgattcactgaaactttccgtttcaatcaatatcagtcccagcataataaattacctcattcaatcactcaactctgcattatctgaagctggaaagctcacaaacctgttctccaatggcggaaatgcaaaaattgttgaaacacagagagggacaatgcaagattgcaaggaactacatgaaatcacactatcttccttgaaaaaatcagtatcaagaattcaatcaggtgattcgaagaagctaggtgatgcaagatcttatctaagtgcagctctcacaaacaagaacacatgtctagaaggattggattcggcttctgggactttgaaacctgttttggtgaaatcattgacgagtacttacaagcacgtgagtaactccctttcaatgattcccaaatcgggtggtggcaaaaaaggccatagaaatcgtcgcctgttggggtttccgagctggttgggacggagagctcgccggattttgcagagcagcgacgatgaatacgatccgagtgacgtgctcactgtagctgccgatggaacagggaatttcagtaccataaatgatgctataaactttgctcccaacaatagttatgacagaatcataatctatgttagggaaggggtttatgaagaaaatgttgaaatcccaagctataaaaccaacattgttcttctcggagatggaaatgatgtcaccttcattactggaagccgaagcgtacgtgatggctggaccactttcagatctgcaactgttgcggtatctggcgaaggctttttggctcgggatataacaatcgacaacagtgcaggccctgagaagcaccaagcagttgcattaagagtaaacgcagatttcaccgcattatataggtcttccatcaatggtttccaggacacattatacgtccactcttttcgacaattttaccgtgaatgcgacatatcaggcaccatagattacatcttcgggaacgcagctgttgttttccaagcatgtaacataatcactcgaatgccaatgcccggtcagttcacagtaattacagcacaatctcgagactccccagacgagaccaccgggatttcgatccaaaactgttcgattcttgccacggttgaactgtatgggaattcaaaccgcttcaaaagctacctagggaggccatggaggatgtattccacaacagcttatattgaatcatatattgatgattttataaatcctagtggatggactaagtggtccaacgatgaaggctcagacacactttattatggagagtacgataattatgggccaggctcagggacggatagcccagtcacttggcctggatatcacgtaatggaatatgatgttgcttataacttcactgtttcggagttaattaccggtgaagcttggctggattctactgctttcccatatgatgatgggattcgacaaaataacttcactattcggtatttctaacactgcagtcgtgaaagataatgtacataagggaacttttactttacaggtcagaccccacatggatctccactctttccgtggagggggccgc

>PMECon5 (Gh/GbPME5)

tagagcactaatatcccacaataaaccccaattaaacctttcgaaacctattacaattcacacattggtgagccatccatggcattggcacgtcttcaaatcctcgtagcactttccttggtgcctgtttttctttttccggttactttaggatatagggctaatgatgtgagatcatggtgcaggaaaactcctaacccacagccgtgcgagtattttttgagccatgatcccaaaaaaaccccaatcaaagatgagttccaatttttcaaaatctccacccacctcgctctagagcgtgccgcacgagcagaaagtaacacgcattcattgggcactaagtgccggaatgaacgtgaaaaggctgcatggtccgattgcgtaaacttgtatgagttaaccatccttcgtctcaacaaaactgttgattccggcaccaatctcaataaggatgatgctcaaacatggctcagcacggctttaaccaatctcgaaacatgccggaccgggtttatggaactcggggtcccggattatcttcttcccatgatgtctaacaacgtttcccagttaattagtaacaccctggctctcaacaaggcaccttacaaggaaccaacctacaaagatgggttcccgacatgggtgaaacccggtgacaggaagctgttgcaatcgtcgtcgccagcttctacggcgaatatcgtcgtggcccaagatggttcagggaattataagacgataaaagacgctatatccgctgcttcgaagaggtccggaagtggaaggtatgtgatatacgttaaggcaggcacatacaaggaaaatgttgagataggaagcaagctgaaaaacatcatgatggtgggcgatggtatagggaaaactataatcacagggagcaaaagcgtcggaggaggatccacaacgttcaattcagccaccgttgccgttgttggagatgggttcattgctcgtggcattacgttcagaaacaccgctggtcctactaaccatcaggcagtggctctacgttcaggctccgatctctcggtgttctacaaatgcagcttcgaagggtaccaagacactctttacgtccattccgaaaggcaattttacagagaatgtgacatctacgggaccgttgattggatattcggcaacgcagccgttgtgtttcaaaactgcaacatatacgcacgcaatccccctaataagaccaacacggtgacggctcaaggccggaccgacccaaaccagaacactggaattataatccacaactccagagttacagcagcttccgatttgaagccagtgcaaagctcggtgaaaacatatctagggaggccatggaaacaatattcgaggacggttttcatgaaaacgtatctggatagcttgattaacccggccggctggatggaatgggatggtaattttgcacttaagacgctgtattatgcggagtatatgaacactggccccggttcatccacttctaatagagtcaaatggggaggctaccatgttcttaagagcgcaagcgaggtgtcaaagttcaccgtcggtaactttctggctgggaattcttggttgccgagcaccggtgtgcctttcacttcagggctttaatttgttgcgatttcatagttaatttaattgcttgtaaaataagtaacagggtttaatagggttaaggctgtataaaccctcaatattgtacctcggccgcgaccacgctaagccatcgattctttgtgtaacgttgtaaaatctttgttaatgaataataaagttgaatttcatcc

>PMECon6

ggaaaaaatatcggaaagattgaagttggtgccataatcagcacgattctcctccttgctcgacttgctgtgtcacaaaatccagcagagatacccgcagataaatcccgagtgaatgcttggttcaatgccaatgtcaagcctgcctcagcaagagttggcaccatagaccccgcccttggtcaggctgaggctgaacctaaagtcattaaagtggtacaaggtggtggcggagatttcgacaccataaccaaagccattgaaagtgttcccacaggaaacaccaaacgtgtgatcatatcgattggacctggagcttatcgggagaaaatcaaaattgaacgaactaagcctttcattacattgattggagatcccaaaagtatggcaaatttgacattcgacggcacggctaagcagtatggaacagtagctagtgccacccttattgttgagtctgatttcttcgtggctgctaatctgtttatagtaaacactgctcctaagccagacggggaaatggagggagcacaaggggtttccttgagagtttctggtgataaagcagctttctataattgcaagatcattggtttccaaaatactttgtgcgatgacaagggcaaccatttctttatgaattgctacattcgtggaactgtcgatttcatttttgggaatgggaagtctttatatctgggaactgaattatacgtggaggaggataagagacaaacagtaattaccgcacaagatagggaaaataatgaacaggatcacactggtttttcatttgtgcaatgcaaaattacagggacagcccaaggtgcatatctaggtagagctgggaagagtagtccaagagttgtttttgccttcactgatatgagcaacgtagtccatcccgagggatggtctcataatttaacagcagagcgtgcccagactcttttctatggagagtacaagtgctcgggacttggggcagcttcggctgcgcgagtgccatactcttcgcagctaactgagacggttgctttacgattcttgactcttggcttcattgacggaagcagatggttgcttccgccgccaaatctaaagatggataaaactattgttttattaggcacacatgcacagctatatgaatagtctgttgatccagcagcatgtatttccatgcaaagcctttgtgtaatttttgttgcttttttccttttattacttataataaaacaggctgtggcgtagaaaaggataacgttatagaattattctttaagaaatgacacaa

>PMECon7

gtattattcaagctcaatattcaagctcaagaggaaactacttcgggaaaaatcaatggattcgacgcctaacgttgtggtcgctaaggatggtagtgggaaatatgattgcatcacagaggccttagctgaagtcccgttgaatagttgcgatcgatttgttattcacattaaggctggtacttataaggaaaaaatcctcgtgaccaaggagatgactaatgttatgttccttggtgatggccccactgagaccatcatcaccaactgcgttaattgttgtaaggataatgttaaaacattcgacactgcaactgttggtgtggatggggctggtttcatggccaagtgcgttgggttcgataactcagcaggacccgaaggtcatcaagcagtggcttttagggcatcctgtgataaggtcgtcatgtttaattgtcatttcactgggtaccaagacaccctttacgctcatagagacaaacaattttatagggattgtctcatcagcgggacggtggacttcattttcgggaatgcggcgagtgtgttccaaaactgtcagatcgtcgtgaggaagccaggtgagaaccaacacaacatggttacagcacacggaaagaaagaagaagaaactaacacagctattgtgcttcaaaattgtaccatctcgggcgcccctgactacctcccggtgaaggacaagaacaagacgtaccttggtcgtccctggaaacagtttgcgacaactgtcataatgcaatgtcaaatcgatgacatcattacaccagagggttactcccccatggaaggcaccgtaggattggacactggttattttgccgagttccaaaataggggaccaggtgccaacaccgagggcagggtcacatggaaagctatcaagaaaatcgatatggatgaagccatgaaatggactcctcgtgtctttttaaaatccgacgaatggctggcacaaactggcgttccctttgatcctgatatgacccctggagtttaaactttcaaaaccatcgccattagtcttcttttattatcattattatttgaaataatgttgtcgattattaattaatccgaacaaccttttcctatttttgtaataataataataatatattcaactcttattgaaggc

>PMECon8

acctctaataaaggctcatgcaatctcctcctcttcccctcaaagcattccaaaatatgtctccaataaaccttgcatttgcttctctattgatcgtcgcgatctatttcccgtgtttcgccaacggcgatgataaggaccccgtgaccgtcctcgatgcccctttcttgacgaagaaaatcaacagcaatgtgacgatcaaagttgatatcaacggcaaaggggactacacgtcgatccaagaagcaatcaatgccgtcccgaaagggaattctaaatgggttgttatccatgtcaagaaagggatttatagagaaaaggttcatataccaaaagataaacggtatatcttcatgagaggcaatgggaggggaaggaccgctattatatggtccttgagttccgtcgataataaggcctccgccacatttaccgtcgaagccaagcatttcgtcgccttcggaattagcttcaagaatgaggcacctactggtgttgcgtatacatctcaaaatcagtcagtggcggtatttgtgggtgcagatagggttgcattttaccattgtgctttctttagcacccacaacacattgttcgattacaaaggccgacattactaccataattgctacatccaaggctcgattgatttcatttttggccgcggaaggtcgttgttccacaattgcgagatcttcgtcttgcaagacaagaggattgcgatccacggatcgattaccgctcaaaacagggcacaccaagaggaccacagcggattcgtgttcgtcaaaggcaaggtttatggcatcggaggtgtttatttaggaagggctaaaggaaaatattcgaccgtggtcttcgcacaaacatacttgtccaggacaatcgtccccgcaggatggaccgactggagctaccgcggtggccctgatcatctgttccatgccgaatacaagtgtcacggtccgggagccgatacattcgggagagttcattgggcgaaacaattgacggacgaggaagctaagtactggacttccattgagttcatcaatggcaacgtttggcttcctgcatggctttgaacagaagatacatattttgcaatgaggggaaaatttatggaattagtttatttaaaaaaaaaattgatttgtattgtatgagaggagaactttggatgcattcaatgtatagtttcataagagtctaatgaaaatgaataataccatttc

>PMECon9

aagaacttgtcaagtcttttatcaccaaaacaactgatgagcttgacaaggctttcaccaaagccacgcacgttcgaattgaaaaccgatgtggagagaaaagcgtttgatgtttgtaaagaagtgatggccaatgccaaggaagaattacagagttccgttgatagagtaggaacaatagaaccagggaagttaccaagcaatggtgatttgaacacttggttaagtgctgtaatgtcttaccaagaaacctgcattgatagtttccctgatggaccattgaagaatgatttcaggactacccttaactctagccaggtttacactagcaattcccttgccatggtcagacaattatcttctttgatgactttaggtaaggaaaagccaccagccaaacgccgtcttttacaaaccaagtttcctttgttggccaagggtgggtatccgagctggtttaaccatgaagaacgcaggctgttgaaggaagcagatgatgacaagaagccaattcccaatgtaacagtggcacaagatggtagcggaaatttcagaactattaatgaagcacttgctgctgtgccacaaaaatacgagggaaggtatgtaatatatgtcaaggcaggaatatacgacgagactgtaatcgttccaaagaaaatggtaaaccttaccatttttggtgatggatcacaaaagagcattatcactggagccaaaaattttgttgatggattccccacttaccagacggcaagttttgtggcttctggtcctggctttatcgcaaaaagcatgggatttagaaacaccgctgggcctgataaacatcaagcagtggccgcaagagtcgatggtgacagggccattttcctcaactgtcgtttcgaaggattccaagacaccttatacacacaaactcaccgtcagttctaccgaagttgcgtgatcgccggcaccatagatttcatcttcggcgacgcgacagtcgtgttccagaactggttgatttatgttaggaagcccaacgacaaccaaaagaacatcgtcaccgcccaagggaggaaagaaaagttagaaacaacaggnatcggtatacaaaacagtaaaatattaccagaggaaccattcaaaccgctcgcaaaacaattcaagaattatctaggaaggcatggaagggatactcgaggaccatctaatgggaaaactggatcgaggatttcatggaccctgctggatggcttgaatgggaagngacttttgcactaagaaccctttttt

>PMECon10

gaagaaaataaacaaattactctttttattaaaaggaaagaagcaggaggaggaaatggatggaaaaagaatcgagggcaatgaagtttatgctttagcaatgtgtgcaagtattctcctctttgctccgattgttgtgtcacaaccaataccggcagataaatcccaagtagaagcttggtttaatggcattatcaagccggtgaaggaaaggggtacaaccttagaccctgaattggttcaagccgagacagaacctagaatcataaaggtgatgcaaggtgggggtggagaattcgataccataaccaaagccatcgagagcgttccatcagggaacgccaaacgcgtgattatatccatcggacctggatcttacaaagagaaaattagaattgaaagaaataagcctttcattacattgttaggagatcctaaaaacatgccaaatttgacatttgacggcaccgccaagcaatatggaaccgtagatagtgccactcttattactgagagtaattactttgtgggtgctaatctcaatatagtgaactctgctccaaggccagacgggaaaatggtaggagcacaagcggttgctctgagagtctccggcgacaggtcggctttctataactgcaagatcattggcttccaggacactttgtgcgatgacaggggcaaccatttctttaaggattgccatattcgtggcactgttgatttcattttcggaagcgggacatctttatatctgaacacggaaatattcgtggtaggagatccagaaggagatccggaaatggcaataattacagcacaagcgcgagaaagttcatcggaggacacgggttattcgttcgtgcatggcagaattacaggaacagcgaaggacgtatttttgggcagggcctggaagagcagtccgagggttgtttattcttatactgaaatggacgaaatcgtccatcctggtggatggtctagtaatcgccaacctgaacgagccgacactgtgtactacggggaatacaaatgcacggggaaaggcgcgactcctgctacacgagagaaattcgtcaaacaactatcggatgcggaagctaaaccattcttggttcttgactacgttgaaggtaccaaatggttgcttcctcctccgacagtacctaaataattcattcattcccaactttttcatgccggcaacatatcctcacagtattgctaattcaagcagactctcgtctacctctttgctgggtggaaataactagccgtccattttattttattttttttttcccttcccacggcttgtattttaacaaatagccaaatgtcaataagaagcagaatttagtcttatttttattaatagtaaggatgttgatatttgccattatgtagaaaatttccatcatatcaagttaaaattatgt

>PMECon11

tgataagcaataagcaactccagaacttcaaaaaaggttttcttctttcttgctttcttttcttcaggggaaagaaagagagaagaagaaacttctttttgaaacaaaaaaagttgtttaaaaatggcgaataatgctattattggtatttgtgcggtgttcctggtggccttggtggtagccgtggtggtgggtgttacgcacataaaaaacaagagcgatggtgaagagatatcgagttcgaacaaagctgtgcaagccctatgtcaacccacgaattataaagagacgtgccagaaaagcttggcgtcatcaaattctagcgacgttaaggagctgataaggacaggtttccaagccgggctagtcgagataaaagaacgtgttagcccattcagtgacggtccaagagttgatcaaggatgagaacaacaaagcggcactctgttgtttgccaagaggtgttggacttagccattgatgactttcaaaagtcattcgacatgctaggggaatacgacatgagcaagatcggaaaataccttttggaactaaagacctggctaagcggtgcattcacgagtcaacagacatgtatagactcattcgcggaatcaagtaacgaatcatcgcagaagatgcaatcaatcttgaagactagcatggagattactagcaatgctttagcgatgcttaatgggttatccaccatcgtgaaggaacttaacattccaaacgtcggcaacatcgacaccacggaggtcaatcgtaagcttttgtcggctgaggatatgcctgagtggattagtcaagctgaccgaaaactccttcaagcaaaaccaatagatttgaagcctaacgttgtggttgctaaagatggtagcgggaaatatgatactattaacaaggccttggctgaagtccccgtgaaaagtcccgatcgatttgttattcacattaaggctggtacttacaaggagaaaatcaacgtgaccaagcaaatgactaatgttgtattcatcggtgatggcccaaccaagaccatcatcacaaatgacattagcgtcgctaagaatccaccggttagaacataccgcactgcaactgttgctgcggatggggctggttttatggccaaggacattggattcgataactcagcaggacccgaaggtcatcaagcagttgcctttagggccactgctgatagggtcatcatgttcaactgccatttcactgggtaccaagacactctttacgctcacagagagagacagttatatagcaactgtctcatcaccggaacggtggatttcatcttcggggatgcggcgagcattttccagaactgtatgctcgtcgtgaggaagccagggccaggccaaaactgcatggttactgcacaaggaaggaatgatcttggaacaaactcagccattgtgcttcaaaactgcaccatctcgggcgcccctgattacatcccagtgaaggatacgaacaaggcgtacttggggcgtccttggaaacaattcgctaggtccatcataatgcaatctaggatcgacgacatcattcaaccagagggttatgcccccatgacaggcaccataggaatagacacttctttcattgccgagtttggaaacaggggacccggtgccgataccagccgtagggtagcatggaaaggtatcaaaaagatagatataaatgaagccaacaaatggactcctcgcgtctttctcgaatccgagacttggattccgagttccggcgttccctatagtcccgacatggtccctggagtttaagcagcttattatcgataccaacgccattagtatgcttattatcatatcattgttctaattatgtcatttttttactaattaattaaacagtttttttaatcttttatctatctatgtatatatgttgaattctttggtttcctgattgttgtttccattcacttattaatgatgtttgcaaaccccaaaaattaagtcaacgtttaaatattattgaacttaat

>PMECon12

acaaagaaaacaaacatattactcttattctataaataaaaaaggaaaaaaaaagtaaatggaaggaaaaagaatcgagagcagtgaagtttatgttgcagcaatgtgtgtaagtattctcctctttgctccggttggtgtgtcccaaccaataccagcggataaatctcaagtaaatgcttggtttaatggcattatcaagcccgtgaaagaaaggggtaacaccttagaccctgaattggttgaggccgagacagaacctagaattataaaggtgatgcaaggtgggggtggagaattcgataccataaccaaagccatcgagagcgttccatcagggaacaccaaacgtgtgattatatccatcggacccggatcttacaaagagaaaatcagaattgaaagaaataagccttttattacatttttaggagatcctaaaagcatgccaaatttgacatttgatggaaccgccaagcagtatggaaccgtagatagtgccactcttattactgagtgtagttactttgtgggtgctaatctcaatatagtgaacactgctcctaagccagacgggaaaatggtaggagcccaagcggttgctttgagagtctctggtgataggtcagctttctataactgtaaaatcatcggcttccaagacactttgtgcgatgacaagggcaaccatttctttaaagattgccatattcgtggtactgttgatttcatttttggaagcgggaaatctttgtatctgaacacaaaaatatttgtggaaggagatccgggacttacagtaattacagcacaagcgagagaaagttcatcagaggacacgggttattcgtttgtgcatggtagcatttctggaacagcgaagaatgcatttttgggcagggcctggaagagcagcccaagggttgtttatgcatatactgaaatgggcaacgttgtcaatcctgctggttggtctcataatctccaacctgaacgagccaaaactgtttactatggagaatataaatgcacggggcaaggcgccaatcccaagacacgggagccattcgtcaaacaactaccggatgcagaagctcaaccattcttggttcttgactatgttgaagctaccaaatggttgcttccttctccaacagtacctaattaattcattcattgtcaacttaatcacgcaagtagcatatcttcgcaatattagaaattcaagtagaccctcatttaccttttcgccgggtggaaataactagccttctatttcattttatttttccttccaaaggcttgtatttttaacaaataggaagttttcaataagaagcagaattcattattatttctatt

>PMECon13

ggaaaattttatgattttttgagggtttgttaagaataaatagaaaacaatggcgaacaatgttgctgttattggtatctgttcggtgttcttggtggcagcagtggtggcagcagtggtgggtgttacgcatttaaaaaacaacggtgattcgaaaagcggtgaaatatcgagttcgacgaaagcggtgcaaactgtttgtcaacccacacagttcaaagaggcatgcgagaagagcctggagtcatctaattccaccgaccctaaggagctgataaggacgagtttccaagcagcgatagaggaaataaggaaagtgttggcgaattcagcgacgatacaagacttgaataaagatgacaacaacaaagcagcccttaaggtttgtaaagaagtattggatttatcaattgctgacctgcaactttcatttgataagctggggggatacgagatgagtaagatcggtgattacttactaaacctgagggtgtggttaagcggtgccttaactaccctacaaacatgtgtagactcatacgcggaggtaaataaagaacaagcggaaaagatgaattcaattttgaagacttcaatggagcttacccaaaattctttaaccatggtgactaagttatccaccatcttgaatgaccttaacattccaggcatcaatatcgacacaactgggtttgaacgtaagctcctgtctaacgatggacctgagtggatgggccacgctgagaggagactacttcaagcaaaaccaacagatttgaagcctaacgttgtggttgctaaggatggtagtgggaaatatgataccatcaccaaggccttggctgaagtccctccaaaaagccccaatcgatttattattcacattaaggctggtacttacaaggaacaagtccttttacctaaggagatgactaatgttatgttcatcggtgatggtccaactaagaccatcatcaccaatgaccttaactgcattagggatcatccgcttaaaacattcggcactgcaacggttggtgtggatggggctggtttcatggccagggacattggattcgagaacacagcaggaccccctggtcatcaagcagtagcttttagggcaacatgtgatatggtcatcatgttcaattgtcatttcaatgggtaccaagacaccctttatgctcacaaacagaaacaattttatagagattgtctcatcagtgggacggtggacttcatattcggggatgcggcgagcgtgttccaaaattgtctgatcatcgcgaggaagccaggtgaaaaccagaacaacatgattacagcccacggaaggaaattcccatacactcactcagccattgtgcttcaaaattgtaccatctcgggagcccctgactacctaccggtgaaggacaagagcaagacgtaccttggtcgtccttggaaagctttggcgaggaccatcataatgcaaagcaatattgatgacattattacaccagagggttacacccccatggtaggcaccgtaggagtagacaccggttactttgtagagttccaaaacaggggacccggtgccaaaactgaaggcagggtcaaatggccagctatcaagacaatcgatttaaatgaagccaagaaatggactcctggcgtctttcttgaaactgaaactgataaatggatttcgcaaaccggcactccttgttatcctgacatggtccctggagtgtgaacttccaaaaccatcgccattagtattcttttataatcattattcgtaatcatgttgtcgattattgattaagttaaacaaaattttcctatgttttatttatattaataatatattcattattatgg

>PMECon14

ggggccataagtgataagcaataacctaccccacaacttcaaataaggttttcttctttctcgctttcttttcttcagggggaaagaaagagagaagaagatacttttttgaaaaaaaaaaagttgtttaagaatggcgaataatgctattattggtatttgtgcggtgtttctggtggccttggtggtagccgtggtggtgggtgttacgcacataaaaaacaagaatgatggtgaagagatatcgagttcgaacaaagccgtgcaagccctatgtcaacccacgaattataaagagacgtgccagaaaagcttggcgtcgtcaaattctagcgacgttaaggagctgataaggacaggtttccaagccgggctagttgagataaagaacgtgctagcccattcagtgacggtccaagagttgatcaaggatgagaacaacaaagcggcacttggtgtttgccaagaggtgttggacttagccattgatgactttcaaaagtcattcgacatgctaggggaatacgacatgagcaagatcggaaaataccttttggaactaaagacctggctaagcggtgcattcacgcgtcaacagacatgtatagactcattcgcggaatcaagtaacgaatcatcgcagaagatgcaatcaatcttgaagactagcatggagattactagcaatgctttagcgatgcttaatgggttatccaccatcgtgaaggaacttaacattccaaacatcggcaacatcgacaccacgggggtcaatcgtaagcttttgtcggctgaggatatgcctgagtggattagtcaagctgaccgaaaactccttcaagcaaaaccaatggatttgaaacctaacgttgtggttgctaaagatggtagcgggaaatatgatactattaataaggcattggctgaagtccccgtgaaaagtcccgatcgatttgttattcacattaaggctggtacttacaaggagcaaatcaacgtgaccaagcagatgacaaatgttgtattcgttggtgatggcccaaccaagaccattattacaaatgacattagcgtcgctaagaatccaccggttaaaacataccgcactgcaactgttgctgcggatggggctggtttcatggccaaggacattggattcgataactcagcaggacccgaaggtcatcaagcagtggcctttagggccactgctgatagggtcatcatgttcaactgccatttcactgggtaccaagacactctctacgctcacagagagagacagttatatagcaattgtctcatccccggaacggtagatttcattttcggggatgcggggagcattttccagaactgtatgctcgtcgtgaggaagccagggccaggccaaaactgcatggttactgcccaaggaaggaatgattttggaacaaactcagccattgtgtttcaaaactgccccatttggggggcccctgattacatcccagtgaaggatacgaacaaggggtacttggggggtccttggaaacaattcgctaggtccatcataatgcaatttgggatgggggacatcattcacccagagggttatgcccccatgacgggccccataggaatagacccttttttcattgccgagtttggaaacaggggacccggtgccgatcccacccgtagggtagcatggaaaggtttcaaaaagatagatataaatgaagccaacaaagggactcctcgggtttttttggaatccgagacttggattccgagttccggggttccctatagtcccgacagggtcccgggggtttaaacagctttttatggatcccaaccccattagtatgcttattatcatatcattgttttaattaggtcatttttttactaattaattaaacagtttttttaattttttatctttctaag

>PMECon15

caaagatgagcaataaagtggttgttgcccgtgtttctatcattcttgtggtgggagtcgctattggccttgttactacaattaaccattctcgtgatgctaagggtaaattgtctcctgaaatgaaggttgcgactagcatttgtgcacctactcattacccagatacttgtcagaagactttcgactctatgaacagcactgatcctaaagaattcgttaaaaaggctatctatgctgctgaagaagaagtcaaaaagttcctcaacttttctaactccaagattgctgaagctaaggacaatggcctcacaaagatggccttgaatgattgtaaggacatgatgcaatatgccattgactcacttgaggcaacatacgctgacgtagatggaagtgatttgcataacattgatgatcgcataaacgattttaggacctggttgagcgctgttatctcataccaacaatcatgcttggatggttttgaacatgatcgcaacttgaaagaaaccatggaagaggctattgttgcttctagtgaacacgcagctaatgctttgacaattgtgacaaagttgatcgaaattcttaccaagttaggttccgagttggctagtcctgacactcgtaggcttttttctgttgaggaaacaaactatccttcatggttttcaactacagaccgcaagcttatagctaagattgataatagcaatttgaaaccaaatgctgttgtggctaaggatggtagtggccaattcaagaccattgctgaagctttagctgccgctcccaagaactccataaacagacatataatctacatcaaggctggaatttatgatgagtacatcaccgtggacaaacaatacaccaacatccttatattcaatgaggcaccaagaagaactattgtcgccggtcgtaaggctgtaaaagatggcggtggaattaccacctggcaaactgccactttctcggctattggaaatgggtttattgccaaatccatgggattccaaaacaatgctggtcctgaaaagcatcaggcagtggcacttcgaattcaatcagataagtcggcctttttcaattgcaggatggatggttaccaagacaccttgtacaatcacgcaaaccgccaatttttccgtaattgtgtcatctctggaactgttgactttatctttggcgactctcctactgtaatccaaaactcgttgttaattgtgaggaggccaatggaaggtcaatctaatatggtgactgcacagggtaagacacggattgatgagaataccggtactgtgatccaaaactgcaggattgtccctgagcaaaagctcttcatcgacagatttaagttcccgacatacttgggcaggccatggaagccgtactctacaactattatcatggagtctacgttaggagacttcattaggcgtgaaggatggatgccctttgctactcaaaatcatgaagataccctctattatgcggaatacaataatcgtggccctggtgctaacctcgatgcaagagttaattggaaaggttaccccaagatagacaaagcaacggcgatgaaatttactgtcgaagcattccttcatagcaaagaaaattggttgccttcaactggtattccgttcactgctggattaagatattgaagacattgaaataaaaaaatcatggccaaagaaagaagtttatggtgttatctgctgaaaatctataattttacactataattgttggtctgtgattcctttttacactataattttagcgaatcaacaaattttgaatcttcatcttagaagttgatacgaaatttgctcatttaaattttcatcgcttatgattttgtgtaattagggaaaacagtaagatcaaaataatgaagaatatttttctttaactcaaattatatcttggttatcaacttttttcttttaactttctacaatatgtattttaaatcttgggtctattttttcattaattatgttcaattttattttt

>PMECon16

tgaatgagctccattctcatttaactacaatacccagaatcaatcaatgtccttcaagcagtcctggcctctttactggcaattgaaaaatgataatgaggctattcatgtttatcttagtcttgggtggtgcaagttttgaagtaacctcaaacaggcttagccaatatgaggtgattgaaaaagactatatgaactgggttaaacaaaagtcttcgttcaagcactctctcttcggaaaacccaagaacaaattaaagccctgcttaaccataagagtaaacaaaaaaccaagtcttgcggagtttgccacagtgaaaaaggctattagttcgattccagtcgttaatcactgtagggttgtaatctccattggagcaggaatttacagagagaagattgaaatccctgcaaccatggcttacattacaatagtaggtgctggtgcagacaaaaccgtgattgagtgggatgacacagctgacaaaatggggcagtcaggccattcactgggcacctatggttctgcaacattcgccattaattctccatatttcattgcaaagaacatcaccttcaagaacaaagcaccattaccgccgtcaggtgcattaggaaagcaagcagtggcactgaggatatcagcagatgcagcagcctttatcggttgcaagtttattggagcacaagatactctttacgatcacattggcaggcactacttcaaacactgctacatccaaggttcagtagatttcatattcggaaatgggctttccctctataaacactgccacttgcatgccgtaaccaacagctatggagccttgacagcccagaaaagggaaagcatgcttgaggaaacaggcttctccttcgttaaatgcaaggtcacggggtcgggcgccctctacctcggccgggcttggggcgttttctctagggtggtctttgtttatacttacatggacaagatcatcacccctagaggatggtatgattggggagacaaaaatagagaaatgactgtgttttacggacagtataagtgctcaggacctggagctgaatttggtgggcgagtttcatgggcgagagaacttacacgacaggaggcaaaaccgtttatttcagttgatttcattgatgggcatagttggctttcggttttatgaggatacgtatattcttcgagctctagtttataaaccaggtagttcttcaagtcacccgacaatgtatttatcctcttaaaacggaagaataattccaagaacgggataaatatttatttaccattgatttcccaaaaaaaaaaaaaaaa

>PMECon17

aagggtctaagctctctccttctcctactctctgttcttttactagcatcgaacatgtatcatcctagtgcaagatccaagaccttgcttgttcttttacccatttcagcacttgtattggtcttgctttcgttcgtagctcttctaacaaacagccccagagccacacaactccagcatttacacgtccacaatcacctccaaattgctcactcagcatgtgaagggaccttataccccgacctttgcgtctccaccatctctgttcttccagatctcgcttccaaatcactgccggagctcatccaagcaactctaaaccaaacaatgtacgaggtccgtctctcctctgccaactgcaccggcattgaaaagcggatcaaaagttacagcaaacgggaagaggcagcgataaacgattgcctagagctttttgatgacaccctggaggagctgaaagttgcccttgctgatctcgccccaaagaaattagcagtctccaggaactaccacgacttgcagacatttctaagcgccgcgatgaccaaccagtatacgtgtcttgatgggttcgcacgtagcaaaggaaatgttaggaacattatcaaaaagggtcttcacaacatcactcatcacgtgagcaactctctcgtaatgctcaaaaaagtccccggtgtcaacaaatccaaatccgagtattttcctcaacagggacgcttgaaaaacgggttcccttcgtcgttatcccgccaagatagaaaacttctacaggcttcagttaatgaaaccgagttcgacctcatagttgccaaggatggcactgggaacttcactaccatcaacgacgctgttgccgctgctccaaataatagtaacacaaggtttgtgatatatataaaagcagggtcttattttgaaaatgtagaggtggtaaagaagaagaaaatgctgatgtttgtgggagatgggatcggtaagacagtggtgaaggccaacagaaatgtcgttgatggctttactactttccgttctgccaccgtcgctgtggtgggggagggattcatagcgaaagggataaccttcgagaactcggcggggccgagcaaacaccaagcagtggctttaaggagtgggtcagacctctctgcattctacaaatgcagcttcgttgcttaccaagacactctctacgtccattcccttaggcaattctatcgggaatgtaacatatacgggacagtggacttcatattcggaaacgcagctgttgttttccaggactgcaatttatatgctcgtaaacccaatccaaaccagaagaacattttcaccgctcaaggaagagaagaccctaaccagaacactgggatttcaattctgaactgcaagatcgctgcagctgcggatttagtccccgtaaaatcatctttcaagacgtatttggggcgtccttggaaagagtattccaggacagttattatgcgttcgtacattgatgattcagtggatcctgccggatggttagaatggaatgggacgtttgctttgagtaccctctattatggggagtacctgaaccgagggccagggtccaatacgagtgcaagagtaacttggcctggttatagggtcatcaacagttccactgaggcaactcagtttactattgagccatttatacaaggaaatgagtggctaaattctagcaatattccattcgctctcggcttgaattgagagctaaatgccacttacgtgcctcccttattttatttttcgttaataattggattcctgtgtttgtctgatcaagttagccaccatgcagtttgatcccatgtcaaattgcttttgtactgaaattaataagaaaatgaataattcttttctctcttcctagg

>PMECon18

aattcggcacgagttttgttacaagcaatggcttcgaagctgatatgctctattttaatgatatgtcaccttgttcttcttcctttgttcatatatccttcctttgcagatagtctcccatcggatccggtctcccccggatctgtttgcaagtccaccccagacccttcttattgcagatccgtgttaccaaatcaaaccaccaatgtgtacaactatgggcgtttctctgttcgtaagtctctgtcgcaatccaggaaattcgttaacttggtcgagaagtacttacggaagtaccgccaatcgttatccgtgtccgccattcgtgctttggaggattgccggtttctagctgacctcaacatggatttcttgttgagctcttttaagaccgtgaatgccactagttcaactctgtccaatgtcgaagctgacgacgtacaaacttttttgagtgctattttaactaatcagcagacctgtgcggacgcctcaaaatcaacttcttcagcttggagcataaggaatggcatctcggttcctctcacaaatgacacaaagctttacagtgtttcgttggctctttttactaaaggatgggttcctaagaggaagaagaaggctacgtggcagcctagtagcagcaagcaactaggctttaaacatgggcgtttgcttatgagaatgtcgagccgagcacgctcgatttatgagactgtaagccgaagaaagcttcttcaaacgaacaacactgatgaagaggtcttggtgagtgatattgtgacggtgagtcaggatggtagtgggaatttcactaacatcaatgatgctatatctgcggctccaaataacaccaatggtgcaaatggttactttgtaatttacatcactgctggtgtctatcaagagtacgtttcaattgctaagaacaaaaagtacttgatgatgattggtgatggtatcaatcagacaatcatcactggaaaccgtagtgttgtcgacggatggacaacatttaattctgcaacttttgctgtggtggctccaaactttgttgcgataaacatgacatttcagaacacagctggggcaatcaagcaccaagcagtcgcacttagaaatggagccgatttatcagcattttatagctgtagttttgaaggatatcaagatacgctatacactcattccttaaggcagttctacagagattgtgatatctacggcaccgttgatttcatatttggaaatgccgcggtagttcttcagaactgcaacatataccctcgacaaccaatgagcggccaattcaatgccatcacagcacaaggccgtactgatcctaaccaaaacacaggcatatcgattcatgactgcaatatcatgcccgctgatgatctagcctctagcaataccacattcaaaacgtatttggggagaccatggaaggagtattcgaggactgtatacatgcagaatttcatgggcagtttgatagatcctgctggttggagagaatggaacggggattttgcacttagtacattgtactatgccgagtacgacaatagtggaccgggttcgaacacttcgagtcgggtcacatggtctggttatcatacgattaatgctactgatgctgctaattttacagtgtcgggattcttgttgggaaacgattggttaccgaatacgggagtgccttacaccgcaagcttactagcatgagcaactaattggaaaatgaaaaattcgttctttttttttttttaaattgatttttagattatataataaatgtaaacttaagcatgcaatatcctcgtaagtgtaatgtttacagcttacgttaactgataccaataatttcaaattttaaaagagagcaagactgctataagtctatttcgatcgttctttgtaaagcgcggcc

>PMECon19

aaggggcatcaagtaataaacagcccccacagcttcaaactaagttcctttctttctttctttctttttctgactttctttcaagggaaagaaaggggagaaagattttatgctttttttattataaaataaataaaagaaatggcgaacaacgttgctgttattggtatttgttcggtgttcttggtggcagtggtagtggcagtagtggtgggtgtgtcacagactcatacaaaagaggagagtgattcaaagagcaatagtatatctagttcaaataaagcggtgcaggcggtttgtcaacccacacatttcaaggatgcttgtgagaagagccttgcgtcatctaattccaccgacactaaggagctgataaggtcgagtttccaagcagcgatagaggaagtaaggaaagtgttggcgaattcaacgacgatccaagacttaaataaagatgacaacaacagagaggcgcttaaggtttgtcaagaagtgttggatttatcgattgatgacctgcaattgtcgtttgataagatgggggaatacgatatgagtaagatcggtgattacttactgaacctgagagtgtggttaagcggtgccttaactacccaacaaacatgtgtagacacattcgcggaggtaagtaatgaacaagcggagaagatgaaattagtgctgaagacttcaatggagcttactgctaatgctttaaccatggtcactaagttatccaccgtcctgaaggaccttaacattccaggcctcgatggcatcgacacgactgggttcgaacgtaagctcttgtctaatgatggacctgagtggatgggccatgctgagaggaaactacttcaggcaccgattattaagcctgatgttgtggttgctaaggatggtagtgggaaatatgacaccatcaccaaggccttggaagaagtccctaagaaaagtcccaatcgatttgttattcacattaaagctggtatttacaaggaaaaaatcaatgtgaccaagcaaatgactaatgttatgttcatcggtgatggcccaaccaagaccatcatcaccaatgacattaactgcattaacaatcatccgctcaaaacatttcaaactgcaacagttggtgtggatggggtcggtttcatggccaaggacattggatttgagaacacagcaggacccgaaggtcatcaagcagtggcctttagggcaactagtaataaagtcatcatgttcaattgccatttcactgggtaccaagacaccctttaccctcacaaagggcaacaattttatagggattgtgtcatcagtgggacggtggacttcatctttggggattcggcaagcgtgttccagaattgtttgatcatcgtgagaaagccaggtgagaaccaaaacaacatgattacagcacctggaaggcaatacatagacactgactctgcccttgtacttcaaaactgtaccatcacgggtgcccctgattaccttcccgtgaaggacaagagtaagacgtaccttggtcgtccctggaaacccttggcgagagccatcataatgcaatctacgattgaagacatcattacaccagagggttatgcccccatggaaggcaccaaaggattggacactgcctactttgctgagttcgaaaacaagggacctggtgccaaaactgaaggcagggtcacatggccagctatcaagaaaattgatataaatgaagccaagaaatggacccctggcgtctttcttggatctgaccaatgggttccacaagccggcattccttattatcccgacatggtccctgtagtgtaaactttcaaaaccatcgccattagtcttattattattattattattattattattattattattattattattattattattattattatatatcccctcctcccatatgagaataaaagggaaaattcatcattattatggaagttgcaatatgtaattattttcttattatagaattaaaagtaataatatgttatcggagat

>PMECon20

ctcaaccgctctaacgccgccgccctccgtttaagatcctttctaattctgattaccgcataagatcccccaatgccgctctggcacgtggcaagatcaaggacgctcgtgcgtggatgagcgcggccctttgctaccaatacgattgttggagcgcgctcaagtacgtaaatgacaccaaattggtcggtgaaacgatggcgtttttggactctttaacgcaacacagcagcaacgcgttgagcatgatggtcgcctacgataatatcggggaggacatagccgcgtgggtccctcccaagacggaacgggacgggttctacgagaacgggtcgggtgggacggagctggggtttaacgggggtttaccgtcaaacttgaagacggacgtaacggtgtgtaaagacgggagcggagggtgttacaagacggtgcaagaggccgtcaacgcagcaccggcaccggcaaatgcagagagtacacgccgtttcgtgatatatataaaggaaggtgtgtacgaagaaacggtgagggtgccactggagaagaaaaatgtggtctttttgggggatggaatgggtaaaaccatcattgcgggtgctttaaatgcagggatgcctggacttaccacttatgagactgctactgtcggggttcttggggatggatttatggccagtggactcacaatccggaacacagcaggccctgatgcccaccaagccgtagcctttagatcagatagtgatctttctgtcattgagaactgtgaattcctaggcaaccaagatactctctacgctcactccctccgccagttctacaggaagtgccgtattcaggggaacgtggacttcatcttcgggaattctgcctcggtgttccaagattgtgaaattttggttgctccccggcaggtaaaacccgaaaaaggtgagaacaatgctgtgacagctcatggtagaactgatcctgctcaatcgactggtctggttttccagaactgcttgatcaacggaactgatgaatacatgagatactattatagcaagcctaaagtgcacaagaactttttgggaaggccatggaaggaatattcaaggacagtttttataaattgtgttatggaagcacttattaatccgaatggatggatgccatggaagggcgactttgcattgaaaacactcttctatggagaatttgggaattctggtcttggatccattccattgaacagagttccatggagtactcaaattccaccccagcatgtacatacattttcagtccaaaatttcattcaaggagatcaatggattccaacatcatcttgattccccccaggaatcatgttgattgcaaagttaagatgataagaattccaagaatgtaaacccaaggaaagtttagctaaacatgatcatattcatctgtaacaaaatcttcattggagaatgaagggttagtgccttagcctacgctattttgtccttttcatttttcctaaattatctgtatatacatatttgaagtttgggtgtgtaatggaatatgtttctcagaatatatagaaaaacatcaaaaattgttt

>PMECon21 (Gh/GbPME2)

acagctactacaaagtactgaaataataagcctttatccccttctcttctactaattggcttggctgccattgaaatggctttctccggtgagcgcaagaaaaagcttttcttggctctctttgcgtcaatccttcttgtaactgccatagtcaccattgccaccaccgtctccatttccaaaaagaaatccagtaatactgtagcagctcactccatcatcaaatcttcatgtagctccacgttgtacccagagttatgctactcaacaatctcttcagcaccagatgctgagaccaaggtcaagaaccccaaggatgtgattgaattgtcgttgaacttgacggtgactgctgttcagagtaactatttgtccatcaaaaagctcattagtacccaaaggaagagcctcacggagcgcgaaaaggctgcccttaacgattgtcttgaactagtggatgagactttggatgagctattcgtagctgaacatgatctcagtgactatccaagctttaacaagtcaatttcccaacatgctgatgaccttaagagtcttcttagtgctgcaatgaccaaccaagaaacttgccttgatgggttttctcacgataaagctgataaaaaggtgaggcaagcgttgcttgacgggcagatgcatgtttttcatatgtgtagtaatgccctggcaatgatcaagaacttgacggacacagacatggcaagccaaggttatcatccatcatctgggaggcaacttgaggagcaagaccaaacagaatggcctaaatggctgtcggagggagataggagactgttacaggctacaacagtgattcctaatgtaacagtggccgctgatggtagtggagacttcctcacggtgtctgaggcggtggcggctgcaccggagagaagcaccacgaggtacattattaagattaaagctggagtttatagggaaaacgtggatgttccaagtaagaaaaccaatctcatgtttgtgggagatgggagggtcaacaccatcatcacagctagcagaaatgttgtcgatggcagcaccactttccactctgccactgttgctgcggtaggggacgggttcttggccagggatataacatttcagaacacggctggaccatcgaagcaccaagcagtggcactgcgtgtgggctctgatttatcagcattctacaggtgtggcattttagcataccaggacactctctatgtccacagccttcgccaattctattcacaatgccttgtagcaggcagcgtggacttcatattcggaaatgcagcagcagtgttgcaagactgcgacattcatgctcgtcgacccaatccaaaccaaaggaacatggtcaccgcacaaggccgtagtgacccaaacgagaacactgggattgtgattcagaaatgcaggatcggtgcaacctcggatttagaagccgttaaatccgattttgaaacttatttagggagaccatggaagacacattcgaggactgttatcatgcaatctgttataagtgatattattcatcctgctggttggttcccatgggacaaagatttcgcactcgacactttgacgtatcgggaatatcagaatactggccctggagctaacacgtcaagcagggttacatggaagggttatagcgtgatcaccaacatatcggaggcacaaacctatactgctcggaattttattgggggagctaattggttaagcgccacgggctttcctttctctcttgatctttgaataaaagctcaagttcagattcgaaataagggaatcatctcctagttttcatttgctcttgctgacattgaatcaacttgttccttgagcatattagtttagcaataataagttattcggtaggtaaatgcatgaatgtacctgaaagaaggcagtatgtgtagttatttcttggattaaaactaaatggattaaattccgagcaatcgagtttgaatattatcaa

>PMECon22 (Gh/GbPME4)

atttgcctttaaatcttcttcactatatagtaatctcatttcttcacctctaagagtttaccgatacatatatgtaacatcttcaatctttgtttcccaaaattaccatgccaaagccattccatttgttggtattcttccttgttgctctttgtctatgcaccaattcaaatgccagcagtactaccaatgagttccttgaaactgagtgcttgaaagtaccagcaactgagtttataggttctttgaaaaccaccattgatgctatacgaaaagctacctctgttgtttctcaatttggtggcttttttcatgattttcgtctctctaatgccatttctgattgtcttgatctgctcgactcttctgctgatgaactaagttggaccatgtctgcttcccagaatcctaatgctaaagataacagtactggtgatctaagttctgatttaagaacatggttaagtgctgcaatggttaaccaacagacatgcattgatgggtttgaaggtacaaatagcatggtcaaaactgtagtatccggtagcttgaaccaaatcacttcactggttcgtaatcttctcatcatggtgcaccctggtcctaattccaagtccaacggtacccgtaatggcagccaaaaaggtggtggtggtgggggtcatccaggccagagccggtttccggtatggttcaaaagggaggatcggagattgttgcaaataaatggggtaacagcaaatgttgtggtggcagcagatgggagtgggaatttcacacgcataatggatgctgtggagacagcaccagataagagtatgaacagatatgttatttacattaagaagggtttatataaagagaatgtggagatcaagaagaagaaatggaaccttgtgatgatcggtgatggaatggatgtcactgtaatatctgggaatcgtagcttcattgatggatggaccacatttcgatctgcaacatttgctgtaagtggaagagggttcatagcaagagacataacattcgagaacacagcaggtccccaaaagcaccaagcggtagcgttacggtccgactcggacctctcagttttcttccgatgtgcaatcaagggataccaagactcgctctacacccacacgatgcgtcaattctacagggaatgtaagatcaccggcactgtagacttcatattcggcgacggcgcggttttgttccaaaattgccaaatcttagccaagcaaggcttaccaagccagaaaaataccatcaccgcgcaaggtcgcaaagacccgaaccaaccaactggcttctccatccagttctgcaacatctcggccgatacagacttattaccttcagttaactccactcctacatacttgggaagaccatggaaactatattcaaggaccattatcatgcaatcctacattagtgatgccataaggccacaagggtggcttgaatggaaccaagattttgccttggatacattatactatgccgagtacatgaataatgggccaggggctagccttagtgagcgagttaagtggcctggttaccatgttttaaataactcagctcaagctgttaattttacagtagctcaattcattgaaggagacttgtggttgccgtcaactggtgttaaatacacttcaggatttggggtataaatatataaatataactttatttatgtattttccgtcaaccggtgttaaataccttctgcaatctaatatatctttaattcccattctctctgcactaacaaaatatattagatttgtagataattattttgtattgcataagatagagtttatatacactttatgtaggagagacaatgtcatattctcccataatgctg

>PMECon23 (Gh/GbPME3)

acttcctctttaattccttcaccttcctcccagcataactccccactctctcctccataaatatttatttacacctctctctccctgcgtcttcttcacgacatgctatccttttttctataatttccaagtacatttcttcattctttttttattgcctataaacatgccaaaattttcctcaccctcttcctatatcttgctcttctcgcttgccctttgtttctgctccagttccaatgccggtagcagccatgaatttccggtaaatgtgccacctgctgagtttgcaggctctctaaggaccaccattgatgctataagaaaagtgatccctatcgtttctcagtttggcagcttttttggtgattttcgactttctaacgccatttctgattgccttgatctcttggatttttctgctgatcaactaagctggtctctgtctgcttcccagaatccaaatggtaaacacaacagcaccggtgatgtagcttctgatttaaggacatggttgagtgctgcaatggcaaatcaggagacatgcattgaagggttcgagggtacaaatggcatcgccaaaacagtagtagcagggggtctgaaccaagtcacttcactggtcagtgaccttctcaccatggtgcaaccaccaggttccgattccagatccaacggtgacaggaaagttgcagagaagaaccggttcccatcatggttcgaaagggaagaccagaaactgttgcaagcaaacggggtaacagccgatgctgtggttgcattagacgggacagggaccttcactaacataatggatgctgtggcagcagcgcctgattacagtatgaaccggcatgtgatttacatcaagaagggtttgtataaagagaatgtggaaatcaaaaagaagaaatggaacctgatgatggttggtgatggaataaatgggacaatcatatcacggaaccgaagcttcgttgatggttggaccacatttcgatcagcaacatttgctgtaagcggaagaggattcatagcaagggacataacatttgagaacacagcaggaccccaaaaacaccaagcggtagcattgcgatcggactccgacctatcagttttcttccgatgtgccattaagggctaccaagattcactctacactcacacaatgcgtcaatttttccgagaatgcaagatcactggcaccgtggatttcatatttggtgacgcctccgtagtattccaaaactgtcaaatcctagccaagcaaggcttaccgaaccagaagaacacgatcaccgcacaaggccggaaagacccgaaccagccgacagggttctcgatccaattctgcaacatctcagctgatgcggatttgttacctttcgttaactctactcctacataccttgggagaccctggaagctgtattcaaggaccataatcatgcaatcctacattggtaatgctgtaaggccacaagggtggcttgaatggaaccaagattttgctttggacacattgtattatgctgagttcatgaattttgggcctggggctggccttggtggcaggattcaatggcctggctaccatgcattgaataactcggctcaagctggcaattttacagtggctcgactgatcgaaggggacctatggttaccgtcaactggcgttaaatacactgccggattgggagcataaataagcaatcaacgcagcttcaatacttcatttatgtttttctaatataaaatccctttcatataacactagagaggtttttttcttttttcttttgtttagattcttttttttaatctatcagaaatgatgaatgaaattattaaagattacattta

>PMECon24

atcatgtaagtagttgtagtagtatttatagcttttatcttcctctcattactgacccaaattgaaaaaaagaaaagatcaatgggatacgacttactccgacgaccggaatttgaaagttccggtgatcaccggcaacaacgtgaagaagaaggagaagaacccagacctgacaacaagaagaacaggcttgttatcgtttcggttattgctttgggtatgatcgttgtgtcagctgtttgtgctgggcttgcaatcgtgctccgtgaaggagctgccgaccgtgttggcggcggcggtggaggaggcgtattggtccaccggaaacccactcgagctatttccaagacgtgtagtaaagctcggttcccgagtctctgtgttacctcactcctcgagttccctggctcactcgctgctagtgagcaagaccttgtccacatttcgttcaatatgacgttacggcactttagtcaagctctttatacgacagcgtcgatctcctacgttcaaatggatccacgagtacggtcagcgttcgatgactgcctcgagcttctagaagattccgtcgacgcactctcccgttctctctcctccgtcgtgccttcgcaagacggcggttataaaggcagctcgtcccaagacgtgatgacttggctgagcgcggccttgacgaaccacgacacgtgtacggaggggttcgagggagtgagcgggacggtgaaggaccaagtggcggcgaaactgaacgacttggcggagctcgtgagtaattgcttgtcgattttcgctgcgagcggcggagacgatttcggcggagtgccggtgcagaacaggaggttgctggcgtcgggtgacgatttatcgggagaaaatgtcgacgaggacaaattcccgaagtggctagggaggaaggagagggtactgttgaacacccccgtgtcggcgatccaggctgatataatcgtctcgaaaaacggtaccgttaagacgatcagcgaggcaattaaaaaggcgccggagaatagtactcggcggatcatcatttacgtgagggcaggaaggtacgaagagaaagatttgaaggtggggagaaagaaaataaacttgatgttcataggggacgggaagggtaaaacaataatttcaggtggaaaaagtatttttgacaacatcaccactttccacacggctgcctttgctgcaaccggggccggttttattgcaagagacatgacattcgagaactgggccggaccagctaagcaccaagcggtggctcttcgtgtcggggcagatcatgccgtggtttaccgttgtaacatcatcggataccaagacactctctacgttcactccaaccggcaattctaccgggaatgtgacatttatgggacggttgatttcattttcggtaacgccgccgtggtattccaaaactgtagcatttatgctcggaaaccgatggccttacagaaaaacaccatcactgctcaaaaccgaaaagacccgaatcaaaatacgggcatctcgatccatgcatgtcggattctaccagcatcggatctcgtcgcatccaacggtagctttgagacgtacttggggcgtccgtggaaattgtactctagggttgtgttcatgttatcatacatgaataatcatgtaccccccaagggatggctcgagtggaacaccacattcgccctggatacactatattacggtgaatacatgaattacgggccaggagcggcgatcgggcaacgaatcgggtggccgggatatagggttattacttccgagattgaagcaaataaatttacagtccaacaatttatttacggatcatcatggttaccatcaactggagttgctttcttggctggtttacaagtttaaagcttgaatgttgtttcccattttgtatttattttagtttggtccatctatccccaactatttttttatatatattttaatcaatttaataattctaaattctaaataaaaaaaaaaaaaaaaaaa

>PMECon25 (Gh/GbPME1)

cgtaatgcaaaaaagaatatgcgaattacctgtgctgagtgtgcactcaaagctctttaaatcctccaaccaggctgccgcttctcttcacagttcacccttccaacttctcttctttgtagccaaaaagaaaatatgagccgaattaaagaaactttatccaacatttctaattctgccaaacacatttccttcaccaagaaacacaagaaaattttcttggcactatttgcatctttagtcattgttgctgctataatcggcattgttgctggagtgagctcacggaataactccgatgagtccgatacttcccatcatgccgttgtgaaatcagcttgtagtggtacgttctaccctgatttatgtttctcggctgttactaccgtccctgctggtactgccaaaaaggtgaggagccaaaaggatgttatcgaattgtctcttaacatcaccaccactgctgtcgaacacaattacttcaagattaagaagctgttggctcgaaaggacttgacgacgcgtgaaaagacggctctccatgattgtttggagactattgacgagaccctcgacgagctccacgaagctgttgaggatcttcatgagtaccccaacaagaaatctttgacccagcacgcggatgacctcaagaccctgatgagtgccgcaatgaccaaccaggaaacttgcttggacgggttttctcacgagggtgccgacaagaaaatccgtgaggtcctgatcgatggcgagaagtacgtcgaaaagatgtgcagtaatgcgcttgccatgatcaagaacatgacggacactgatatagcaaacgagatgatgctcaagtcatcgaacaggaagctgaaggaggacgaaagcggcattgcctggccagagtggttgtccgctggcgacaggcgcctgcttcagtcgtcttcggtgacccctaacgtggttgtggcggccgatggtagcgggaacttcaaaacggtgtcggaagcggtggccaaagcaccggagaaaagcagcaaaaggtatattataagaatcaaagcaggcgtctacagagaaaacgtggaagtcccaaagaagaagagcaacataatgttcatcggagacgggagaactaaaacaatcatcaccggcagcaggaatgtggtcgacggaagcaccactttccactctgccacagttgctgtggttggtgaaaagttccttgcccgagatataaccttccaaaacacagcaggtccctccaagcatcaagctgttgccctccgtgttggctctgatctctcagcattctacaattgtgacatgctagcataccaagacactctctacgtccactccaatcgtcaattttacgttaactgcctcgtagcaggaacagttgacttcatcttcggaaatgccgcagccgtgttccaaaactgcgacatccatgctcggaaaccaaactcagggcagaaaaacatggtgacagcccaaggcaggaccgaccctaaccaaaacacaggcattgtgatccagaaatgtaggataggtgccacttctgatttacagcctgttcgtaaaaacttcccaacatatcttggcaggccctggaaggagtactcgaggactgtggtaatgcaatcaacaatcagtgacgtgattcaacctgctggttggcacgagtggagcgggagtttcgccctgaaaacactgttttatgccgagtaccagaacaccggggctggtgcctcaacttctgcgagagtgaaatggggagggtacaaggtgatcactagtgcaagtgaagctcaggcttttactcctggcaggttcattgctggaggaagttggttaagctctacaggctttcctttcgctcttgggttgtaataattttaaagattgtattgtcaatgtagccacctgccgacttcattgtttggtttgagatttgctttgcggccatgtatctgttaaattattactaaattaatagtagtaataagcgaatgccaaggttttcttcaaatttatcttggtgagacagcttggtttcatgtcaaaaaaaaaaacacaaa

>PMECon26

aatccatctctctcatttaactcttattatattgttgtagtgatattaatagccttttttttgttgctatctacattttgttactgatttttgcagccaccacctgaaaaaggaacagcaatggcgtatgacaggctccgaccatctgaacccggcagctctggagttactcagcaacaaaatcatgaagaacccaaagccaaaaacaacaagagaaagatcgttattctttcggttattgcattgacgatgatcgttgcctcggctgtttgtgccggactcgtgattggtctccgtgaagccggtgctgatcctgtcggtacccaagtccgacgtaagccgacacaagctatttctaaaacttgtagtaggactcggttcccgaacctctgcgtcaactcactcctcgagttccctggctcgctcactgctaatgagcaagacctggttcacatttcattcaatatgacgctgcagcactttagtaaagcgctttatatggccaactcgatctcattcgtccagatggaaccacgtgtacggtcggcgtacgacgattgcctcgagcttcttgatgattccgtcgacgcgctctcccgttccctgtcgtccattattccctcccaggacggcggaaacaatggcggaggatcaccccaggacgtgatgacgtggctcagcgccgcgctgacgaaccatgacacgtgtacggagggattcgatggagtgagcgggacggtgaaggatcaagtgactgacaaactgaaggacttgtcggagctggtaagtaactgcctgtcgatcttcgctgcgagcggcggagacgatttcgccggagttcctattcagaaccggaggctgctatcggcggatgacgatttatcgggagggaatgtcgacgaggagaatttcccgaaatggctggggagaaaagagagggagctgctgaacaagcccgtgtcggggatccaggccgatataaccgtctcgaaagatggaagcggcaccgttaaaacgattgctgaggcgattaaaaaggcgccggagcatagtactcgtcggatcatcatctacgtgaaggcaggaaggtacgaagaggataatttgaaggtggggaggaagaaaataaacttgatgtttataggtgacggaaaaggtaaaacagtgattacaggaggaaaaagtgtagctgagcacatgactactttccacactgctgcctttgcggcaactggggctggttttattgcaagggacatgacattcgagaactatgccggacccgccaagcaccaagcagtggctcttagagtcggagctgaccacgccgtcgtatacaggtgcaacatcatcggttaccaagacactttatacgtccactccaaccgtcaattctaccgcgaatgcgatgtttacggaacagttgatttcattttcggtaacgccgccgtagtactccaaaactgtagcctttacgcccggaaacccatgccccaacaaaaaaacaccatcacagcccaaaaccgaaaagacccgaaccaaaacaccggtatttcaatccatgcatgtcgaatcatacccacacaggacctcgccgccgccaaatcaagcttcccaacatacctaggccgtccgtggaagctatattcaaggaccgtatacatgttatcatacatgggcaaccacgttcactcaaggggatggttagaatggagcggaacattcgctttagatacattatactacggcgaatacatgaattccggcccgggtgcggcggtcggacaacgagtaagatggcctgggtatcgagtcatcacttcagaagttgaagcaagtaaattcaccgttgcaaaattcatctatggaacttcatggttaccatcaaccggagttgctttcttttctggcctacaagtttaatttttttttttacgtcctaatttttttttacccatgatattgtaataaaggtcgttattatcgttaaaaatttaactcccgacatgttctagaaggttagaactagaatatagaatataaaaaattgtgtattgtattttcgtatttttttttaaatgtgagcaatataaacacataaaattacaagtgttaaaaaaaaaaaaaaaaaaaa

>PMECon28

aaaaaaacagcaatgaatcatgtcttctgaaaaacctgtaaattgtttttctatttattgcctcttcgctcgatctcatcagcctctttcttaaagaaaaactcaaatctcactaaaaaccccacaaaacattcaagacagaaaagagggcgtgttgttgttgtttctcttcgacttcgatcgatgtcggcgagggtaataacggtgtcccaagacggcaatggcgactaccgaacagtgcaggaagccattgacactgtcccactctgcaacacctgccgcaccatcattcgactgtcacccggggtttacaagcaacctgtttacattcccaagaccaagaacctcatcaccttggctggtctccgccctgaactcactgttcttacatggaacaataccgctactaaaattcaacatcaccagggttcgaggttaattgggactggaacgttcggttgtggcactgtgattgttgaaggagaagatttcattgctgaaaatgtcacatttgaaaactcttccccagagggttcaggacaagctgttgcaatcagagtaacagcggatcgatgtgcattctataattgcaaattcctgggctggcaggatactctatacttgcatcatggaagacagtacttgaaagactgctacattgaaggaagtgtagacttcatttttggaaatagcacagctcttttagagcattgtcatatacactgcaagtcagctggatttataactgcacagagccggaaaacttctcaggaatcgactggttacgtgtttttaagatgcgtcattactggcaacggagggtcttcttattcatatctcgggcgaccatggggaccctttggaagggtggtctttgcctacacatacatggaccaatgtatcaaacatgtaggttggcataactggggaaaagccgaaaacgagagaagtgcttgcttttacgaatacagatgtttcggccctgggtgctgtcaatcaaaacgggttacttggtgtcgggaactgctggaggaagaagcagaagagttccttatgcacggattcatcgaccctgatccgaaccgaccatggcttgctcaaagaatggcactgaaaataccatattctgcataaaaaagagaaacagatagtggtggtatgagttaataatgaggaatataatggtgtgggttggaaatatttgaaaccaaaaaataagggaagaaggggttaaaaaaaa

>PMECon31

aacaagtgcatcttcgttgcctaccaagacacactctacgtccattcccttaggcaattctatcgcgaatgtgacatatacggcaccgttgacttcatctttggaaacgcagctgcggttttccaagcctgcaatttatatgctcggaaacctaatcccagccaaaagaacattttcacagcgcaaggaagagaagaccctaaccagaacactgggatttcgattctgaacagtaagattgctgctgctgcggatttaataccgctaaaatcatcgttcaagacgtatttggggcgaccctggaaagagttttccaggactgttattatgcgttcctacattgatgattcggtggatcctgctggatggttagaatggaatgggacatttgctttgagtaccctttattatggggagtatttgaacggggggcccgggtcaaatacgagtgcaagggtaacttggcctggttatagggtcatcaatagttccactgaggcagatcagttcactgctggggcgtttatacaggggaatgattggttaaattcaactgatattccattttctctcggtttgatactttagagctaaacgtcactttccttcttttcaagttatttgtatgtcaatctactagaattaattttttattttcaaataaattattttgctttgtttatataactcctgggcaattcatctgagacttgcccttccagtactagtagtgatccccgccttgtctcgtcctctgcctgatg

>PMECon32

aaacgattgcttggagctctaccagaacaccatttcccatcttaaccgatcaaagagcaccaacagtccattagatgctcaaacctggttaagtgctgccattgccaaccatcaaacttgtcaaaacggcttcattgacttcaacttggcctctcatttacaaaccttaccacccatgctagccaacttttccaagttgattagcaattccctggccatcaataagcccactattagtgttaccgctaaacaagttggcaatagacgcctgtttgcccatggattccccacttggctttccggttccgaccgaaagctcttgcaaaaaatcggtgcaccgttaaatgctgacattgtggtggctcaagacggttctggcaacttcaagactatatcagaggcggtggcggcggctggcggtgccaaaagaactgtcatacacgtaaaagctggagtttacaatgagaatgttgatattcaaagatcagctagaaatatcatgttaattggagatggaattggtgccacggttgttactggtaataagaatgctcaaactacgacaactttgcgcacagcaacatttgccgtcgttggtggtggattcatagctcgtgacatcacttttgagaacaccgccggaccccaaaagcaccaagcggtggccctccgctccggctctgatttctcagtcttctaccgttgcagcttcaagggttaccaagataccttatacgtctattcccaacattgtctcgtcctctgcctgatg

>PMECon33

aaaaaaaagaaaatcccaggtttgtttaatgctctattttaatggagttttcatcttcatcttgtttcagttcctttttattattgttttacgtatgtttttgcttatgtcttgcaacgccgccgttagtggtggcaacaggaaatggttcttcttgtaataatactaatacaacgaggcatcaccataaatggattggacccattggtcaccggttgattaccgttgatgttaacgggtcgggtcagttccggtcagtacaagatgccgttgatgctgtaccggagaataatagaaagaatgttatgatcttaatcagccctgggcattacatagagaaggtggtggtgccggcgacgaagccatacataacgtttcaaggtgccggtagagagacaacgtttattgagtggcacgacagagcttgtgaccgtgggtctaatggtcaacagttgcgtacttatcaaacggcttccgttactgtgtatgctaattatttctctgctagaaatattagcttcaagaatacagcaccggctccgaagccgggaatgcaagggtggcaagctgtggcgttccgtatttctggtgacaaggcatatttctcggggtgtggattctacggtgcacaagacactctttgcgacgatgccggacgtcattatttcaagcagtgttatattgagggttccatcgacttcatcttcgggaatggccgttccatgtacaaagaatgtgaattacactccatagctacgagatttggatccatagcagcccatgacaggaaatcaccagatgagaaaagcgggtttgcttttgtaaactgcacagtgacgggtactggcccactttatgtcggtcgagccatgggtcaatactccaggattgtctattcttacacctactttgacaatctcgttgctcaaggcggttgggacgactgggaccatgtcagcaacaaaaataagactgcgttttttggggtttataagtgctggggtcccggagctgcggcggtgcgtggagtatcatgggccagggaactaaagttcgatgaggcgcatccctttctagccaaaagttttgtcaatggtagacactggattgcaccgtgggatgcttaagtccaactcaaaacaacatacaacctattctatacactatgttccatttcctttcgcataattaattcactgattcaattcaanttttcttgcgcatattggt

>PMECon34 ggcacgagcacaaacacacctgtataatgtctaagtttcaatgttttctttacattgtcatccttgttcttctcttgaattcagctcagacattgtgccataccaaaggtataaaacttaaaaggaagcaccatcacaagcaatcaaatgtgacaggaacccatgtttcagaacaaccattcatgcaatgggttaaatttgttggcagccttaatcactcagttttcaggacagcaaaaaacaagcttttcccttctcgtaccattaccgtcgacaagaatcccaaatccggtgacttcaccaaaattcaagatgccattgattctcttcctttcattaaccttgtgagagttgtaatcaaggtccatgctggggtctacacggaaaaggttaacattccaccattgaaggccttcataacaattgaaggagcaggggcagataaaacaattgttcaatggggagacacagctcaaacacctggagctagaggacaacctttaggaacctatggttctgcaacttttgctgtgaattcaccttattttattgccaaacacattacattcaagaacactgcaccaattccagcaccaggagcaatcggaaaacaagctgtagcatttagaatatcggcggatacggctactttcgtcggttgtcgattccttggagctcaagatacactttatgatcactttggcagacattattataaagattgttacattgaaggctcagtagatttcatctttggcaatgctctctccctctttgagggatgtcatgtgcatgcaatagcaaggttaacaggggcagtaacagcacaaaacagagggagtatcctagatgacacagggttctcttttgtgaagtgtaaggtgacagggtcaggggctttatatttagggagggcatggggtcctttctcgagggtggtctttgcttacacttacatggacaacatcatactgcctaagggctggtataattggggtgaccctaaccgtgagatgacggtgttctatggacagtacaaatgcacggggccaggagcaagctttgcaggcagggtttcatggtcaagggaacttacagatgaggaagctaaaccttttatttctcttagtttcattgatggctctgaatggatcaaattataattatacaatgatccatcaatttttatttttttaatgtatatcaacaaaacgagaaagtagtatttgtaaaagatgatagctaggtttttctgaagacgatgatgataatcatgataaatttgattagaagcaattcactttctaaaaaaaaaaaaaaaaaaaa

>PMECon35

gcaaaacaaagactttaatttccggcagccacaactttgttgatggaactcccacatttgctacagctactttcgctgtggcaggaaaaggattcattgcaaagggcatagggttcattaacacagctggtgcagtaaagcaccaagcagtggctatgcggtctggttctgatcgctccgtattctaccgctgtgcatttgatgcctaccaggacactctctatgctcattccaatcgtcagttttatcgggaatgtgacattttgggtacaattgacttcatttttgggaatgcagctgttgttttccaaagttgcaacatcctgcccaggcagcctttggctaaccagttcaataccatcacagctcaaggcaaaaaagaccctaaccaaaacactggcatttgcattcagaagtgttcaataagtgcatttggcaaccttactgctaatacttaccttggcaggccctggaaagaattctccactactgttattatgcagtccaagattggggcgttcttggaccctgtgggctggagaggatgggttgctaatgttgatccacctatctcaattttctatgcagaatatcagaacagtggacctggatcaaccgtggatcatagggtgaaatgggctggttataggtcctctctctcagacgttgatgctgggaagttcacggtggctacgtttatacaaggtcatgattggcttcctgctgccactgtctcgtatgaacctgctttctgattttcttgctgattccaggttagggttgtgatatgttcgaagctcaaatttttatattttttttttatttattctttttttgagcttcaatttgctctgtcagctgggtatattatatttgtgactccattgatatgaattggaggttttggtgtcatgcatttgcattttaaaacatggtgcagaagtcaaataatttactactt

>PMECon37

attcggcacgagtcgataccttcctggctgatgatttgcatgcgttgcttagtgctgttttaacgaatgtgcaaacttgtatcgaagggcttgaagctacaccgtcggcttcgagcattaaaaatggcttattgccatctatttctaatggaacaaatttcctcagtgtgtctcttgcacttttcaggcacggttgggttcatggattaattaaatctctaacaggaagaaaccatgtgttttccaatttggtaaatggcagggactctcctttgcctttaataatgtcggaccatgatcgagcagtttacgaatccgcaagccggcagaagcatgttcgagcagatgaggaagggaaggacaaaggggtttccgtgagtcaagtagtggttgtaaatcctgatggaagtggcaacttcaccactatcaacgaagcagtggccgctgcaccgaataataccggggatagcaacagatactttttgatttacgtggtcgcgggtgtctacgaagagtatgtttccatacctaagaagaagcagaatgtgatgatgattggtgacggtataaacaagacgatnatcactggaaaccgcaactttgttgatggatcaaccacattcaactctgcaacatttgctgntgntggtaagggatttggttgcggncaacattacatttcgtaacacnggncggacctagcaaacaccaagctgtcgcgggtcgaaatggagccgatntgtccacattctaccgatgtagcttcgaaggctaccaagacacttttntgctcacttcctg

>PMECon41

ctggagctcgcgcgcctgcaggtcgacactantggatccaaagaattcggcacgaggaagaacacaatcacggctcaaagtcgaaaagacccgcaccaaaacacgggtatttcgattcatgcatgtaggatcttacccacgccggatctcgcagtaatgaacggtagcttccaaacgtatctagggcgtccatggaagctgcattccagggttgtgttcatgttatcatacatgcatgaccacattgaccctaggggatggctggaatggaatggttcatttgccttagattcactatactatggtgaatacatgaattatggagctggtgcggcagttggtcaacgggtcaaatggcctggctatagagtcatcacttcagaatctgaagcaagcaatttcactgttgcacaatttatttatggaacattatggttaccatcaacggggattgctttcttggctggtctccaagtttaaaattaaattttctttaacctttttcccaaaaatatatttcagaatttgtttttagatatggtaaagtgtcactgttcctgttcattttgaaagtccaaacatgttataggctctaaaagntactttcttttacaaaaaaaaaaaaaaaaa
