# Supplementary material for: Pectin Methylesterase and Pectin Remodelling Differ in the Fibre Walls of Two Gossypium Species with Very Different Fibre Properties
Source: PLoS One. 2013 Jun 5;8(6):e65131. doi: 10.1371/journal.pone.0065131 (PMC3673955; doi:10.1371/journal.pone.0065131)
Supplement: Figure S2 — Comparison between colorimetric and enzymatic methods for quantification of total extractable pectin in cotton fibre cell walls. Comparable results of estimated total pectin extracted from G. hirsutum fibre analysed either by the new enzymatic method described in Materials and Methods and the traditional colorimetric assay of Filisetti-Cozzi and Carpita [46] (DOCX) [file pone.0065131.s002.docx]

**Figure S2.** **Comparison between colorimetric and enzymatic methods for quantification of total extractable pectin in cotton fibre cell walls.** Comparable results of estimated total pectin extracted from *G. hirsutum* fibre analysed either by the new enzymatic method described in Materials and Methods and the traditional colorimetric assay of Filisetti-Cozzi and Carpita [46]
