# Supplementary material for: Pectin Methylesterase and Pectin Remodelling Differ in the Fibre Walls of Two Gossypium Species with Very Different Fibre Properties
Source: PLoS One. 2013 Jun 5;8(6):e65131. doi: 10.1371/journal.pone.0065131 (PMC3673955; doi:10.1371/journal.pone.0065131)
Supplement: Table S2 — PCR primers used for the cloning of the cotton PME cDNAs and for quantification of expression levels by quantitative realtime PCR. (DOCX) [file pone.0065131.s004.docx]

**Table S2.** PCR primers used for the cloning of the cotton PME cDNAs and for quantification of expression levels by quantitative realtime PCR.

| **Gene** | **Forward primer** | **Reverse primer** |
| --- | --- | --- |
| Primers used for amplifying partial cotton PMEs from fibre cDNA | | |
| PME1 | AGAGCAACATAATGTTCATCG | GCAAATCTCAAACCAAACATG |
| PME2 | TGCGTCAATCCTTCTTGTAAC | CAAACTCGATTGCTCGGAA |
| PME3 | ACACCAAGCGGTAGCATT | AACCTCTCTAGTGTTATATGAAAG |
| PME4 | TGTTATTTACATTAAGAAGGGTT | ATGCGCGGTGATGTATTTTT |
| PME5 | ACCGACCCAAACCAGAACAC | GAAGCCTTCAAGGGGATAA |
| Primers used for Q-PCR | | |
| Gh/GbPME1 | CCTCAACTTCTGCGAGAGTG | CCGCAAAGCAAATCTCAAAC |
| Gh/GbPME2 | GCAGGGTTACATGGAAGGGT | CTTCTTTCAGGTACATTCATGC |
| Gh/GbPME3 | GAATGGAACCAAGATTTTGCTT | ATTGAAGCTGCGTTGATTGC |
| Gh/GbPME4 | GACTCGGACCTCTCAGTTTTCTT | TGAAGGTAATAAGTCCGTATCGG |
| Gh/GbPME5 | ACATATCTAGGGAGGCCATGGA | CCCAGCCAGAAAGTTACCGA |
| Ubiquitin | CCAGAAGGAATCCACTTTGC | CCAGCTCACATCAGCATACG |
